# Cysteine oxidation and disulfide formation in the ribosomal exit tunnel

**DOI:** 10.1038/s41467-020-19372-x

**Published:** 2020-11-04

**Authors:** Linda Schulte, Jiafei Mao, Julian Reitz, Sridhar Sreeramulu, Denis Kudlinzki, Victor-Valentin Hodirnau, Jakob Meier-Credo, Krishna Saxena, Florian Buhr, Julian D. Langer, Martin Blackledge, Achilleas S. Frangakis, Clemens Glaubitz, Harald Schwalbe

**Affiliations:** 1 Institute of Organic Chemistry and Chemical Biology, Center for Biomolecular Magnetic Resonance (BMRZ), Goethe University of Frankfurt, Frankfurt, Germany; 2 Institute of Biophysical Chemistry, Center for Biomolecular Magnetic Resonance (BMRZ), Goethe University Frankfurt, Frankfurt, Germany; 3 Institute for Biophysics, Buchmann Institute for Molecular Life Science, Goethe University Frankfurt, Frankfurt, Germany; 4 Max Planck Institute of Biophysics, Frankfurt, Germany; 5 Institute de Biologie Structurale, Grenoble, France; 6 Present Address: Institute of Science and Technology Austria, Klosterneuburg, Austria; 7 Present Address: Centre for Misfolding Diseases, University of Cambridge, Cambridge, UK

**Keywords:** Structural biology, Cryoelectron microscopy, Molecular modelling, Solution-state NMR

## Abstract

Understanding the conformational sampling of translation-arrested ribosome nascent chain complexes is key to understand co-translational folding. Up to now, coupling of cysteine oxidation, disulfide bond formation and structure formation in nascent chains has remained elusive. Here, we investigate the eye-lens protein γB-crystallin in the ribosomal exit tunnel. Using mass spectrometry, theoretical simulations, dynamic nuclear polarization-enhanced solid-state nuclear magnetic resonance and cryo-electron microscopy, we show that thiol groups of cysteine residues undergo S-glutathionylation and S-nitrosylation and form non-native disulfide bonds. Thus, covalent modification chemistry occurs already prior to nascent chain release as the ribosome exit tunnel provides sufficient space even for disulfide bond formation which can guide protein folding.

## Abstract (web summary)

As protein synthesis takes place, newly synthesized polypeptide chain passes through the ribosomal exit tunnel, which can accommodate up to 70 residues in the case of a helical peptide. Here the authors show that oxidation of cysteine residues in the nascent chain can occur within the ribosome exit tunnel, where sufficient space exists for the formation of disulfide bonds.

## Introduction

Cellular protein synthesis takes place at the peptidyl transferase center inside the large ribosomal subunit. The newly synthesized polypeptide chain passes through the ribosomal exit tunnel. The exit tunnel is up to 100 Å long and 10–20 Å wide, with the widest point at the exit site of the tunnel^1^. The ribosomal exit tunnel accommodates 33 residues of an extended polypeptide chain and up to 65–70 residues in an α-helical conformation^2–5^.

Protein folding occurs in a co-translational manner^6^. Current studies in structural biology focus on investigating structures of the nascent chain (NC) within the ribosomal tunnel. Several studies, involving pegylation assays^5,7^, cryo-electron microscopy (cryo-EM)^2–4^ and translation assays^8,9^ have revealed not only α-helical conformations within the ribosomal tunnel, but also β-strand formations for small monomeric protein domains^9^. In addition, fluorescence resonance energy transfer measurements showed that the NC can adopt compact non-native conformations within the tunnel^10,11^. A nuclear magnetic resonance (NMR) study has revealed that the ribosome stabilizes unstructured protein conformations until most of the protein has emerged from the tunnel^12^ and a recent cryo-EM study uncovered dynamic sampling of different conformations of the NC within the tunnel^13^.

Post-translational protein modifications have been widely studied^14,15^. However, modifications of the NC inside the ribosomal tunnel are unknown. The coupling of protein folding and disulfide bond formation after proteins emerge from the ribosome has been studied for the eukaryotic proteins low-density lipoprotein receptor (LDL-R)^15^, β2-microglobulin and prolactin and the disintegrin domain of ADAM metallopeptidase domain 10 (ref. ^16^). In these studies, covalent modifications including disulfide bond formation are generally believed to occur outside the ribosomal exit tunnel.

Here, we show that oxidative modification of cysteine side chains by glutathionylation, nitrosylation, and disulfide bond formation can occur inside the ribosomal exit tunnel during the expression of the mammalian eye-lens protein γB crystallin (GBC). The two domains of this monomeric protein are connected by a six-residue flexible linker^17^ and consist of two greek-key motifs. The N-terminal domain of GBC contains six cysteine residues. Cysteines 18, 22, and 78 are in close proximity in the crystal structure of full-length GBC, and cysteines 18 and 22 are partially oxidized in native GBC purified from bovine eye lenses^17,18^.

In a previous study, we detected pronounced effects of codon usage on the stability and folding of GBC^19^. Different synonymous mRNAs coding for the identical amino acid sequence of GBC led to differences in translation rates. Unexpectedly, they also led to variations in the extent of disulfide bond formation in *Escherichia coli*. In the case of faster translation, 58% of GBC was oxidized in the N-terminal domain, while the more slowly translated protein was fully reduced^19^.

Based on these findings, we here ask whether disulfide bonds in the N-terminal domain of GBC can form co-translationally in the exit tunnel and remain oxidized, even in the reducing *E. coli* cytosol. Disulfide bonds could be sterically protected by the subsequently translated C-terminal domain. Variation in translation speed would therefore result in different degrees of protection and thus different oxidation levels^19^. This mechanism, however, requires that cysteine oxidation and disulfide bond formation occurs inside the ribosomal exit tunnel which has never been reported before.

In this contribution, we integrate liquid- and dynamic nuclear polarization (DNP)-enhanced solid-state NMR spectroscopy, mass spectrometry, theoretical modeling, and cryo-EM to show that oxidation of cysteine residues in the NC can occur within the ribosome exit tunnel in ribosome nascent chain complexes (RNCs). By arresting the GBC N-terminal domain using SecM arresting peptide^20^ (17 residues) we detect glutathione (GSH) and nitrosyl adducts and disulfide bond formation.

## Results

### Production of RNCs with uniformly ^13^C-labeled cysteine residues

**Fig. 1:**
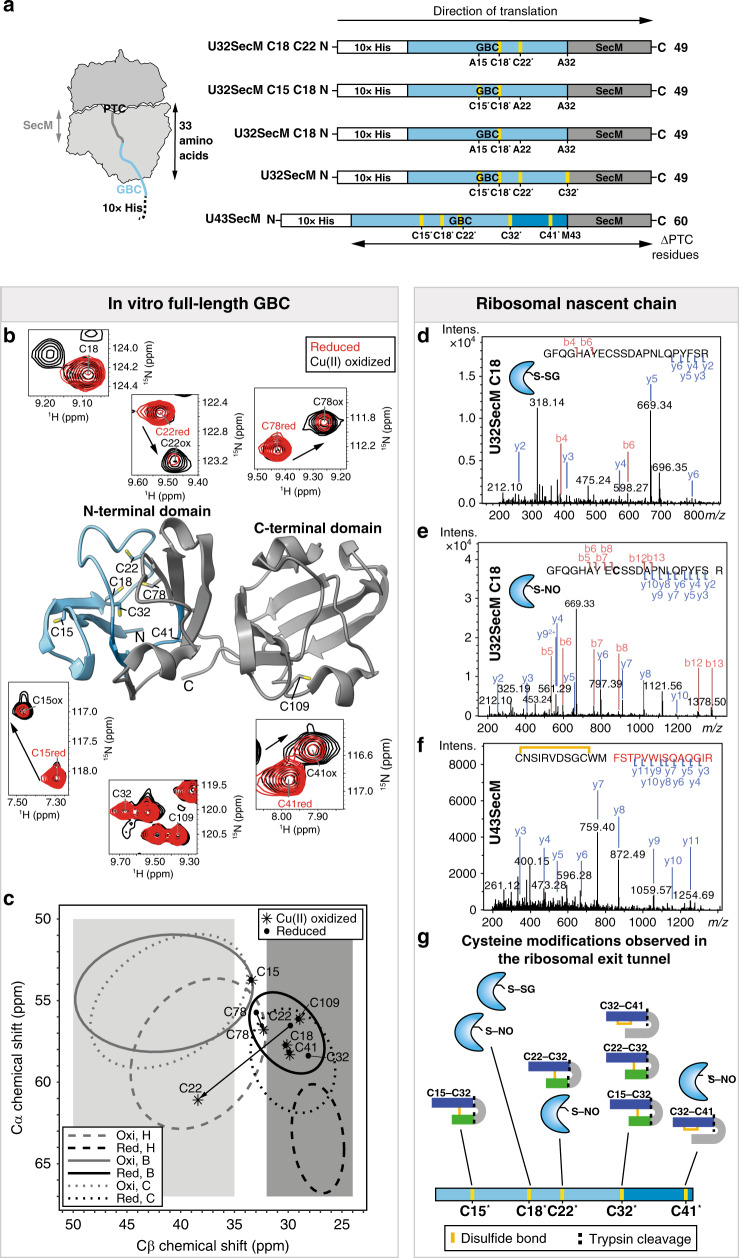
Design of GBC RNC constructs, solution-state NMR investigation of full-length GBC, and LC-MS/MS investigation of RNC fragments. **a** Different lengths of GBC were fused at the N-terminal with a 10x histidine purification tag (white) and at the C-terminal with a 17-amino acid SecM sequence (dark gray). Numbering excludes the N-terminal methionine of GBC. GBC residues 1–32 (light blue), 33–43 (blue). ^13^C-labeled Cys residues are marked with an asterisk (*) and highlighted in yellow. Number of GBC RNCs residues are given from the Peptidyl transferase center (PTC) to the N-terminus excluding the 10x histidine purification tag (ΔPTC residues). **b** Lengths of the RNC construct*s* shown on the full-length GBC structure (PDB 4W9A) and 2D backbone ^1^H-^15^N-HSQC NMR spectrum of cysteine residues of full-length GBC reduced (red) and after oxidation with Cu(II) (black). Cysteine residues not changing position after oxidation (Cys18, Cys109) or that could not be unambiguously identified due to overlapping signals (Cys32) were labeled only once. The complete ^1^H-^15^N-HSQC NMR spectrum is shown in Supplementary Fig. 3. **c** Cα and Cβ chemical shifts of the six cysteines within full-length GBC reduced and after oxidation with Cu(II) compared to values derived from a chemical shift database^24^. Ellipses contain 90% of the corresponding chemical shifts of each group^24^ (H = helix, B = β-strand, C = coil). The spectral region for oxidized C_β_ cysteines is highlighted in light gray, and for reduced cysteine in dark gray. The overlapping region of both states is shown in white. **d** Fragment spectrum of the tryptic peptide GFQGHAYECSSDAPNLQPYFSR (precursor *m*/*z* 695.54 (4+)) from U32SecM C18 with a glutathione modification on Cys18. **e** Fragment spectrum of the tryptic peptide GFQGHAYECSSDAPNLQPYFSR (precursor 835.03 (3+) *m*/*z*) from U32SecM C18 with a nitroso modification on Cys18. **f** Fragment spectrum of the tryptic peptide CNSIRVDSGCWMFSTPVWISQAQGIR (precursor *m*/*z* 980.46 (3+)) from U43SecM with a loop-linked disulfide bond (Cys32–Cys41). Matched fragment ions are indicated by dashes in the sequence and respective labels on peaks. **g** Overview of cysteine oxidation states (*S*-nitrosylation, *S*-glutathionylation, disulfide bonding) of the NCs of all RNCs (U32SecM, U43SecM, and U78SecM and mutants) as observed by LC/MS-MS (Supplementary Table 1). Trypsin cleavage site is shown as dashed line, disulfide bond as yellow line.

We prepared GBC constructs truncated at different lengths (32, 43, 78 residues) with ^13^C-labeled cysteine for NMR studies (Fig. 1a and Supplementary Fig. 1). These constructs are named according to their length in amino acids. For the Cys to Ala mutants only the remaining Cys residues in the construct are given.

RNCs were prepared in *E. coli* BL21(DE3)Δtig strain^21^. NC expression was induced in the presence of rifampicin to suppress incorporation of isotope-labels into ribosomal proteins^22^. RNCs were purified by Ni^2+^-affinity chromatography and sucrose gradient ultracentrifugation^23^ (“Methods”). We obtained 3 mg intact RNCs per liter of expression culture with stalled and ^13^C-labeled Cys residues in the NC after extensive optimization and used those for cryo-EM and solid-state NMR studies. The integrity of the RNC was confirmed by western blot analysis (Supplementary Fig. 2).

### Oxidative modification of native full-length GBC and NC fragments

One of the crystal structures (PDB: 4GCR) of GBC isolated from bovine eye lenses contains Cys18 and Cys22 in two different oxidation states, one fully reduced and one with a disulfide form between Cys18 and Cys22 (ref. ^17^); in the oxidized conformation, the side chain of Cys22 is turned to bring the two sulfur atoms into close proximity. To investigate the cysteine oxidation states in solution, we conducted liquid-state NMR analysis of full-length GBC expressed and purified from *E. coli*. We prepared fully reduced samples and samples mildly treated with Cu(II) to induce oxidation. Our NMR analysis reveals chemical shift and intensity differences between the reduced and the Cu-(II)-oxidized samples in particular for all six cysteines in the N-terminal domain (Fig. 1b and Supplementary Figs. 3 and 4). The ^1^H–^15^N correlation spectrum shows peak doubling for four (Cys15, Cys22, Cys41, Cys78) of the total seven cysteine residues in both domains, and C_α_/C_β_ chemical shifts of Cys22 in particular indicate oxidation. By contrast, Cys109 in the C-terminal domain does not show any chemical shift changes, while for Cys32 this could not be unambiguously detected due to signal overlap. The chemical shift changes are however either the result of direct cysteine oxidation or the result from changes of the backbone torsion angles of remote cysteine residues.

C_α_ and C_β_ chemical shifts of Cys yield information on oxidation state and the secondary structure (coil, β-sheet or α-helix). According to the Biological Magnetic Resonance Bank (BMRB)^24^, Cys C_β_ chemical shifts greater than 35 ppm can be assigned unambiguously to an oxidized state, while shifts below 32 ppm indicate reduced cysteines^25^ (Fig. 1c).

To differentiate the oxidation states of cysteines within the NCs, we investigated NCs using liquid chromatography-coupled tandem mass spectrometry (LC-MS/MS). In the absence of reducing agents during sample preparation, peptides with GSH- and nitrosylation adducts of the NC polypeptides were detected together with intramolecular disulfide bonds (Fig. 1d–f and Supplementary Table 1). We detected GSH and nitric oxide bound to the side chain of Cys18 in the GFQGHAYECSSDAPNLQPYFSR polypeptide from the U32SecM C18 construct (Fig. 1d, e). An intramolecular disulfide bond was detected between Cys32 and Cys41 in the polypeptide CNSIRVDSGCWMFSTPVWISQAQGIR from the U43SecM construct (Fig. 1f). Except for *S*-nitrosylation, none of these modifications was detected in control samples with reducing conditions. In the RNCs comprising the N-terminus most of the cysteines are sensitive to oxidation. Cys32 in U32SecM is involved in several disulfide bonds, notably also between Cys22 and Cys32. The observed disulfide bonds in U32SecM and U43SecM construct are located inside the ribosomal exit tunnel. We then simulated the NC within the ribosomal exit tunnel and investigated U32SecM using solid-state NMR and cryo-EM.

### NC ensemble calculation of U32SecM

To model whether the conformational space within the ribosomal exit tunnel is sufficiently large for disulfide bond formation and whether the NC can populate multiple conformations, we simulated the conformational space of the NC within the tunnel using flexible-meccano^26^, a method that relies on amino acid-specific (φ/ψ) sampling in coil regions of protein structure. The dimensions of the ribosomal exit tunnel were taken from the cryo-EM structure (PDB: 6YS3), and the NC was sequentially built from Ser151 of SecM by adding 26 amino acids to the N-terminal end. Indeed, the ribosomal exit tunnel provides sufficient space for different disulfide bridges to form including a disulfide bond between Cys22 and Cys32 inside the ribosomal exit tunnel close to the constriction site (Supplementary Fig. 5). Further, we were able to observe that the ribosomal exit tunnel modulates the sampling of the NC, with Cys32 and Phe150 showing a higher propensity to sample an α-helical conformation inside the ribosomal exit tunnel of *E. coli* than in solution (Supplementary Fig. 6).

### Solid-state detection of oxidation of NC cysteines

**Fig. 2:**
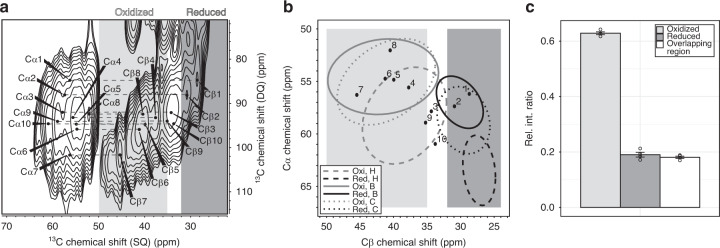
Analysis of ^13^C DQ–SQ solid-state NMR spectrum of ^13^C, ^15^N cysteine-labeled U32SecM. **a** Cysteine C_α_–C_β_ cross peak regions of the ^13^C DQ–SQ spectrum of ^13^C, ^15^N cysteine-labeled U32SecM cryo-EM sample (3.4 nmol). **b** C_α_ and C_β_ chemical shifts from U32SecM compared to values derived from a chemical shift database^24^ (left panel). Ellipses contain 90% of the corresponding chemical shifts within each group^24^ (H = helix, B = β-strand, C = coil). The spectral region for oxidized C_β_ cysteines in light gray, and for reduced cysteine in dark gray. **c** Relative integration ratio (Rel. Int. Ratio) of oxidized (light gray) or reduced residues (dark gray) or the overlapping region (white), which does not allow for differentiation, of one U32SecM sample. Signal intensity of oxidized cysteine residues (>35 ppm), reduced cysteine residues (<32 ppm), and the overlapping region (35–32 ppm) was divided by the total signal intensity of C_β_ chemical shifts. Error bars represent the standard error of the mean and were calculated using four different integration regions (“Methods”). Each circle represents the Rel. Int. Ratio of one individual integration region.

The cysteine oxidation during translation occurs at various sites and is chemically diverse and conformationally complex. To visualize these modifications at atomic resolution, we took the intact SecM-stalled RNC (Fig. 2 and Supplementary Figs. 7–9) and used solid-state NMR to detect the C_β_ chemical shifts of the NC cysteine residues, which are sensitive to the cysteine oxidation states. To tackle the challenges posed by limited sample amount, we applied DNP to increase the sensitivity.

The ^13^C-labeled Cys residues of the GBC NC could be observed in double quantum–single quantum (DQ–SQ) 2D ^13^C-^13^C spectra^27,28^ (Fig. 2a). In these spectra, natural abundance ^13^C signals of other sites in RNC were sufficiently suppressed (Supplementary Fig. 10a). ^13^C ribose signals of the rRNA are visible, due to the highly abundant ribose in rRNA. Isotopic labeling of rRNA bases were not detected. The DQ–SQ 2D ^13^C-^13^C spectra of RNCs show complex spectral patterns composed of many signals, indicating that the NC adopts multiple conformations within the exit tunnel. (Fig. 2a for U32SecM cryo-EM sample and Supplementary Fig. 7 for all samples).

For all constructs (Supplementary Fig. 8), the oxidized C_α_ and C_β_ chemical shifts of Cys residues are spread across the random-coil, β-sheet, and α-helix regions. In the U32SecM cryo-EM sample that was prepared with a higher oxidation ratio, some oxidized Cys residues also sample only the oxidized α-helical conformation (Fig. 2). In the sample with the lower oxidation ratio (Supplementary Fig. 8d) signals in the reduced α-helical area are observed. For both set of samples, we thus detect sampling of non-native compact conformations in the ribosomal exit tunnel.

Integration of signal intensities within characteristic spectral regions provides the relative populations of oxidized and reduced cysteine residues in each construct (Fig. 2c and Supplementary Fig. 9).

Replacement of Cys with Ala in a series of U32SecM mutant constructs (Supplementary Fig. 9) does not significantly change the oxidation level of Cys (16 and 19%). However, the oxidation level is increased to 25% for wild-type U32SecM (Supplementary Fig. 8) and is further increased to 38% in U43SecM (five Cys residues) and 34% in U78SecM (six Cys residues). Even the ^13^C_β_ NMR signals of a construct with a single cysteine (U32SecM Cys18) are indicative of oxidation (Supplementary Fig. 8). in agreement with the results of mass spectrometry.

Thus, the detection of *S*-nitrosylated and glutathionylated cysteine residues by mass spectrometry is confirmed by solid-state NMR as chemical shifts of *S*-nitrosylated and glutathionylated cysteine residues (Supplementary Fig. 11) overlap with chemical shifts for cysteines in disulfide bonds (Fig. 2b).

### Cryo-EM structure of U32SecM

**Fig. 3:**
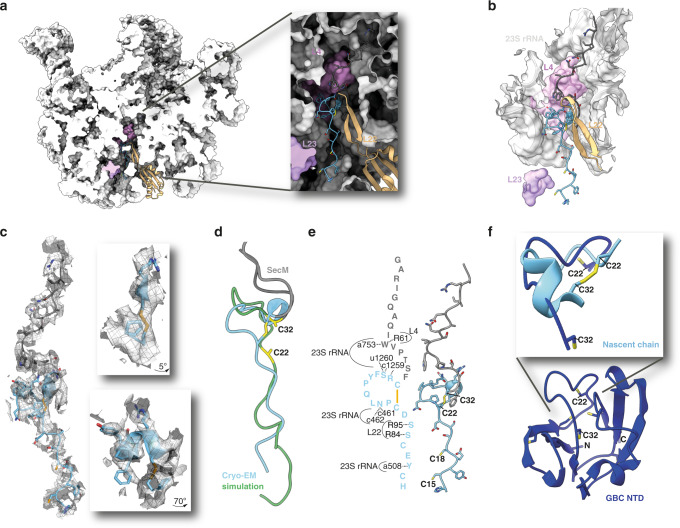
Cryo-EM structure and structural analysis of U32SecM. **a** Side view of ribosomal 50S subunit with focus on the nascent chain. The surface of nucleotides 753, 1323–1325, and 1616 of the 23S rRNA were hidden to gain a full view on the nascent chain. **b** U32SecM in the ribosomal exit tunnel with the surrounding area (within 20 Å) shown in surface and cartoon representation. **c** Cryo-EM density of 2.0 Å around the nascent chain with model of U32SecM and detail view on the α-helix and disulfide bond. **d** Overlay of nascent chain model and flexible-meccano simulation, with possible disulfide bond between Cys22 and Cys32. RMSD of Cys22 and Cys32 to the cryo-EM structure is 3.72 and 2.98 Å, respectively. **e** Model of nascent chain with interactions to ribosomal proteins and 23S rRNA represented. Nucleotides and amino acids are shown in lowercase and uppercase one-letter code, respectively. **f** Overlay of loop between Cys22 and Cys32 of the full-length GBC protein (4W9A) and the nascent chain U32SecM (6YS3). RMSD = 4.33 Å. Cysteine residues are labeled and shown as sticks. The 23S rRNA is shown in light gray, SecM in dark gray, the nascent chain in light blue, L4 in light pink, L22 in light orange, and L23 in light purple.

To determine an atomic structure of the NC containing an intramolecular disulfide bond, we prepared a sample with a higher oxidation ratio of 63% (Fig. 2c). Thus, the shortest construct U32SecM with four cysteine residues was used to determine its cryo-EM structure (Fig. 3b–f). Of 266,000 particles, 210,000 were occupied with one tRNA in the P-site, while 55,000 showed electron density for two tRNAs with the ribosome in a rotated state, indicating two different stalling mechanisms, in agreement with previous reports^20^. We obtained a 2.58 Å (PDB 6YS3) resolution structure for the U32SecM construct with one tRNA bound (Fig. 3a, b) and a 3.19 Å structure with two bound tRNAs using a similar approach as previously described^20^ (Supplementary Fig. 12). The density around the NC is shown in Fig. 3c. A disulfide bond between Cys22 and Cys32 is visible and an α-helical conformation between Phe150 and Tyr28 is found (Fig. 3c). In addition, the two cysteine residues involved in a disulfide bond are located at a similar position in one flexible-meccano simulation (Fig. 3d).

Interactions between GBC and the ribosome involve hydrogen bonding between of Arg31, Ser30 and nucleotides C1259 and U1260 of 23S rRNA (Fig. 3e, Supplementary Fig. 13a and Supplementary Table 6) and between the Asn24 side chain and the phosphate backbone of C461 and C462 of 23S rRNA (Supplementary Fig. 13b). Interactions in the lower region of the tunnel include hydrogen bonding of the carbonyl groups of Ser19 and Ser20 with the side chains of Arg84 and Arg95 of L22, respectively (Supplementary Fig. 13c), and of Tyr16 with the phosphate backbone of A508 of 23S rRNA (Supplementary Fig. 13d). All these interactions potentially stabilize the α-helical conformation of amino acids Phe150 to Tyr28 and the loop formed between Cys32 and Cys22, and bring these residues in close proximity to form a possible disulfide bridge (Fig. 3c). Notably, the residues between Cys32 and Cys22 are also located within a loop in the native structure of GBC (Fig. 3f).

In close proximity to Cys22 and Cys32 are Phe150 from SecM and Arg95 of L22 (Supplementary Fig. 13e). Basic amino acids, such as arginine, in the vicinity of cysteine residues can lead to the formation of Cys thiolate and the high reactivity of this thiolate to further disulfide bond formation^29^. Aromatic amino acids close to Cys residues could undergo S–π interactions and thereby contribute to the stability of the native GBC^30^, which could increase the reactivity of the thiol. The formation of the observed disulfide bond between Cys32 and Cys22 might be enhanced by the tight space inside the ribosome and the surrounding amino acids.

## Discussion

An analysis of 4895 non-redundant human proteins containing disulfide bonds revealed that 298 proteins that function in the cytoplasm and/or nucleus contain 509 structurally defined disulfide bonds^31^. Understanding the coupling between folding and disulfide bond formation is therefore interesting and two models have been developed to explain this coupling: In the folded precursor model, cysteine residues are brought into close proximity after the structure is formed. In the quasi-stochastic model, cysteines pair in an unfolded conformation and influence subsequent protein folding^32^. Although a coarse-gained molecular simulation assumed protein folding guides disulfide bond formation in BPTI, another study showed that co-translational disulfide bond formation in β2-microglobulin in eukaryotes occured only after the entire protein domain entered the ER^33^. Support for the quasi-stochastic model comes from two recent reports: in a cell-free expression system, arrested translation intermediates of the disintegrin domain of human ADAM10 formed non-native disulfide bonds^16^. Further, Robinson et al.^16^ showed that a cysteine oxidation in a disintegrin domain can be rationalized within the quasi-stochastic model, suggesting that secondary structure rather than cysteine density influences the mechanism of cysteine coupling. Non-native disulfide bridges between distant cysteine residues have also been observed in the LDL-R in HeLa cells^34^. The authors of that study hypothesized that co-translational oxidative folding might occur in LDL-R. None of these previous studies, however, address the question, whether disulfide bond formation or cysteine modifications, which can lead to disulfide bond formation, can occur in the exit tunnel within the ribosome.

In this report, we arrested NCs of different lengths and detected oxidation inside the ribosomal exit tunnel, including *S*-nitrosylation, *S*-glutathionylation, and disulfide bond formation. Since the NC was arrested with SecM and further purified, we cannot exclude that the disulfide bond formation occurs after the translation of the NC. *S*-nitrosylation and *S*-glutathionylation are considered post-translational cysteine modifications and can be mediated by *S*-nitroso-glutathione (GSNO) or GSH^35,36^. In general, these modifications are reduced after release of the ribosome within the highly reductive environment of the *E. coli* cytosol with 5 mM GSH and a reduced GSH to glutathione disulfide (GSH/GSSG) ratio of 50/1 to 200/1 (ref. ^37^). Thus, heterologously expressed proteins can typically be produced only in reduced form in *E. coli*, but exceptions have been reported^38–40^. Since the purification buffers did not contain GSH or any nitric oxide-containing compounds, the observed cysteine modifications, however, must have been formed inside the cell and GSH adducts on GBC have in fact been previously reported^41,42^. These modifications could then promote the formation of an intramolecular disulfide. Although it was demonstrated that the NC can be co-translationally arginylated^43^, glycosylated^11^, or acetylated^44,45^, all these modifications require specific enzymes and coenzymes and are thus unlikely to occur inside the ribosomal exit tunnel. We demonstrated that *S*-nitrosylation and *S*-gluthathionylation of cysteine residues can be found deep inside the ribosomal exit tunnel.

We find that the NC adopts compact conformations stabilized by interactions between the NC and the ribosome.

**Fig. 4:**
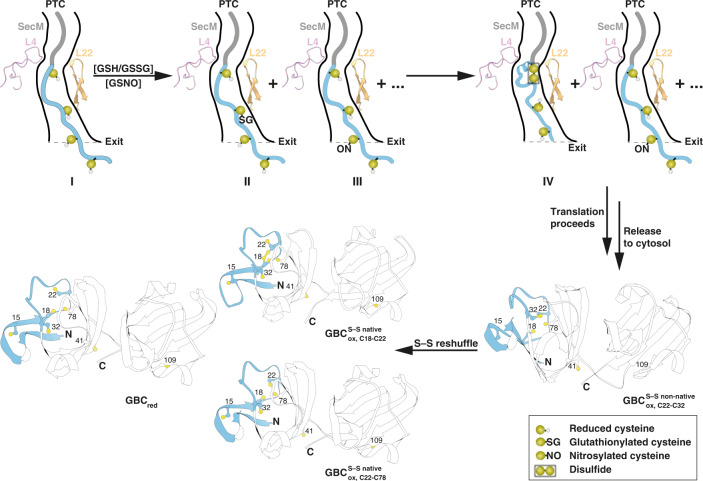
Disulfide bond formation within NCs and after release. Free thiols of GBC nascent chain (I) are modified by glutathione (GSH) (II) or *S*-nitrosoglutathione (GSNO) (III). Such modified cysteine residues with enhanced electrophilicity can be attacked by thiol groups of neighboring cysteine residues to form disulfide bonds (IV). In the released NC, non-native disulfide are either reshuffeled to form native disulfide bonds (C18-C22) or reduced pending on translation rate. SecM is shown in dark gray, the nascent chain in light blue, L4 in light pink, and L22 in light orange. Cysteine residues are shown as yellow ball.

The tunnel dimensions of cytoplasmic ribosomes have long been considered to be universally conserved throughout all three kingdoms of life^46^. A recent study by Duc et al.^47^ revealed, however, a second constriction site and a slightly smaller average radius in the lower part of eukaryotic ribosomes. Simulation of the U32SecM NC inside the ribosomal exit tunnel of *H. sapiens* (Supplementary Fig. 6) revealed differences in the sampling of Arg31, Cys32, and Phe150 compared to the *E. coli* ribosome. Although all disulfide bridges of U32SecM could also be formed in the eukaryotic ribosome (Supplementary Fig. 14). Hence, disulfide formation within eukaryotic ribosomes would require further investigation, since different structures might be obtained. Our results show, however, that the ribosomal tunnel confines the polypeptide chain and allows disulfide bond formation as well as cysteine modifications (Fig. 4). Some proteins or their co-translational folding intermediates could use such early formed disulfides as a means to escape the reducing environment of the cytoplasm. Our work also provides structural and functional information on the conformational space sampled by the polypeptide chain within the ribosomal exit tunnel. This finding has implications for the oxidative folding pathway of the polypeptide chain inside the cell. While part of the chains may already form native disulfide bonds in the tunnel, non-native modifications will first have to be reduced and refold in the cytosol to attain the final native structure. In this respect, our investigations unexpectedly revealed that the detected disulfides in the exit tunnel are different, at least in part, to the disulfides in the native structure of GBC where Cys78 is involved in disulfides. Thus, the disulfide formed during translation has to be reduced and re-oxidized and the early formation of a non-native disulfide will catalyze disulfide bond isomerization once the polypeptide chain is released (Fig. 4). Such catalysis can increase the rate of disulfide bond formation and explain how differences in the rate of translation of proteins expressed from synonymous codons can be preserved in the native state of proteins.

## Methods

### Expression of RNCs

The cells were transformed with the protein encoding plasmids (pET15b, 10x-GBC-SecM derivatives). RNCs were expressed in *E. coli* BL21(DE3) Δtig::Kan cells^21^ for selectively labeled RNCs or in BL21(DE3) cells (New England Biolabs, Frankfurt am Main, Germany) for uniformly labeled RNCs. For uniform labeling the cells were grown in MDG medium at 30 °C until OD_600 nm_ of 5 as previously described^48^. The cells were harvested by centrifugation (2200 × *g*, 4 °C, 20 min) and resuspended in M9 media without nitrogen and carbon source. The OD_600nm_ was monitored and after 60 min ^15^N NH_4_Cl (1 g/l), ^13^C-glucose (2 g/l), 150 mg/l rifampicin, and 1 mM IPTG were added to the medium. After 1 h of expression the cells were cooled on ice and harvested by centrifugation (5000 × *g*, 4 °C, 20 min).

For selectively labeled RNCs, the cells were grown in LB medium at 30 °C according to Rutkowska et al.^22^ to an OD_600nm_ of 1. Cells were cooled on ice and harvested by centrifugation (2700 × *g*, 4 °C, 20 min). Afterwards the cells were resuspended in M9 salts and centrifuged again. This washing step was repeated once more. The cell pellet was then resuspended in one quarter of the volume in M9 media supplemented with all amino acids and ^13^C, ^15^N-labeled cysteine (20 mg/l). The expression was induced with 1 mM IPTG, and 150 mg/l rifampicin was added. After 1 h, the cells were cooled on ice and harvested by centrifugation (5000 × *g*, 4 °C, 30 min). The cell pellets were frozen in liquid nitrogen and stored at −80 °C.

### Purification of RNCs

RNCs were purified as described before^23^ with the exception of the sucrose cushion step. Briefly, the cells were resupended in lysis buffer (50 mM HEPES, 500 mM KOAc, 12 mM MgOAc) supplemented with Dnase I, lysozyme, and protease inhibitor tablet (Roche, Basel, Switzerland). The cells were disrupted using an M-100P microfluidizer (1000 bar, Microfluidics, Westwood, USA). The lysate was centrifuged twice (38,400 × *g*, 4 °C, 30 min) and the supernatant was loaded on a 5 ml Ni-NTA column (Histrap HP, GE Healthcare, Chicago, USA), washed with 4% elution buffer (50 mM HEPES, 500 mM KOAc, 12 mM MgOAc, 20 mM imidazole), and eluted in a linear gradient to 100% elution buffer (50 mM HEPES, 500 mM KOAc, 12 mM MgOAc, 500 mM imidazole).

RNCs containing fractions were pooled and concentrated using a 100-kDa-cutoff concentrator (Vivaspin, Sartorius, Göttingen, Germany) at 3500 × *g* and 4 °C. Approximately 1.5–3.0 nmol was loaded on a 10–40% (w/v) sucrose gradient (35 ml, 25 × 89 mm tubes, SW28 Beckmann Coulter, Brea, USA). The concentration was determined by measuring the absorbance at 260 nm, with one optical unit corresponding to 24 pmol/ml 70S ribosomes^49^. The gradients were spun at 90,192 × *g* (23,000 r.p.m., Beckman Coulter, SW 32 Ti) at 4 °C for 16 h. Afterwards the gradients were fractionated in 1.13 ml fractions using a Piston Gradient Fractionator (Biocomp Instruments, Fredericton, USA). The fractions were loaded on a NuPAGE 4–12% (w/v) Bis-Tris gel at neutral pH with a loading dye at pH 6.8, the purest fractions were pooled, and the buffer exchanged to Tico (10 mM HEPES, 30 mM NH_4_Cl, 10 mM MgCl_2_, 1 mM EDTA, pH 7.5). The final sample was concentrated to 10–20 µM and 14–38 pmol was loaded on a NuPAGE 4–12% (w/v) Bis-Tris gel (Thermo Fisher Scientific, Waltham, USA) for analysis by western blotting. The NC was detected using a Poly-His antibody (Merck, Darmstadt, Germany). The NC was released by addition of RNase A and incubated at room temperature for 10 min.

### Purification of flow-through ^13^C, ^15^N cysteine-labeled ribosomes

The flow-through during the nickel-affinity chromatography of a U24SecM sample was collected and pelleted for 4 h at 225,000 × *g* and 4 °C. The pellet was resuspended in Tico buffer (10 mM HEPES, 30 mM NH_4_Cl, 10 mM MgCl_2_, 1 mM EDTA, pH 7.5), and 1.9 nmol was loaded on a 10–40% (w/v) sucrose gradient. 70S-containing ribosome fractions were pooled and the buffer exchanged to Tico buffer (via dialysis as for U32SecM cryo-EM, for all other samples the buffer was exchanged using protein concentrators). Two hundred microliters with a concentration of 24 µM was loaded on a 500 µl glycerol cushion with 15 mM AMUPol and 30% (v/v) glycerol.

### Growth conditions and purification of ^15^N-labeled ribosomes

*E. coli* (JE28) cells^50^ were grown in M9 media containing ^15^N NH_4_Cl (1 g/l) and 30 µg/ml kanamycin at 37 °C. At an OD_600nm_ of around 0.8, the cells were cooled on ice for 15 min and harvested by centrifugation (5000 × *g*, 30 min, 4 °C). Cells were lysed and purified by affinity chromatography as described before^50^. Briefly, the cells were harvested by centrifugation (5000 × *g*, 30 min, 4 °C) and were disrupted using a M-100P microfluidizer (1000 bar, Microfluidics, Westwood, USA) in ribosome buffer (20 mM Tris-HCl pH 7.6, 10 mM MgCl_2_, 150 mM KCl, 30 mM NH_4_Cl). The lysate was centrifuged twice (38,400 × *g*, 4 °C, 30 min) and further purified using nickel-affinity chromatography (HisTrap HP, Ni-NTA) (GE Healthcare, Chicago, USA) equilibrated with ribosome buffer. The ribosomes were eluted in a linear gradient to 150 mM imidazole. Ribosome-containg fractions were pooled and loaded on a 10–40% (w/v) sucrose gradient. The buffer was exchanged to JE28 buffer (20 mM Tris-HCl, 10 mM MgCl_2_, 150 mM KCl, 30 mM NH_4_Cl, pH 7.6). The final sample was concentrated to 11 µM for the DNP-enhanced solid-state NMR experiments. ^15^N-labeled ribosomes were used to optimize the glycerol cushions required for the solid-state NMR experiments.

### Expression, purification, and oxidation of uniformly ^13^C, ^15^N-labeled GBC

Uniformly labeled GBC was expressed in BL21(DE3) cells in M9 minimal medium (Supplementary Table 2) and purified as described previously^19^. Briefly, the cells were harvested by centrifugation (5000 × *g*, 30 min, 4 °C) and disrupted using a M-100P microfluidizer (1000 bar, Microfluidics, Westwood, USA) in GBC lysis buffer (50 mM sodium phosphate, pH 8.0, 500 mM NaCl, 20 mM imidazole). The lysate was centrifuged (38,400 × *g*, 4 °C, 30 min) and further purified using nickel-affinity chromatography (HisTrap HP, Ni-NTA) (GE Healthcare, Chicago, USA) equilibrated with NTA washing buffer (50 mM sodium phosphate, pH 8.0, 500 mM NaCl, 20 mM imidazole). The protein was eluted with NTA elution buffer (50 mM sodium phosphate, pH 8.0, 500 mM NaCl, 250 mM imidazole) and further purified by size-exclusion chromatography (HiLoad 26&60 Superdex 75 prep grade) (GE Healthcare, Chicago, USA) in IEX A buffer (50 mM Tris, pH 9.0). Protein containing fractions were pooled and further purified by ion-exchange chromatography (HiTrap Q XL) (GE Healthcare, Chicago, USA), which was equilibrated with IEX A buffer. The protein was eluted using a pH gradient over 30 column volumes to 100% IEX buffer B (50 mM Tris, pH 6.0, 50 mM NaCl) The buffer was exchanged to NMR buffer (50 mM Tris, 200 mM NaCl, pH 8.0, 10% D_2_O, 0.1% DSS) and backbone assignment experiments were performed. After backbone assignment experiments, the protein was diluted to 10 µM and oxidized with 2 µM CuCl_2_ under continuously stirring and air supply overnight. The protein was loaded on a gelfiltration column (HiLoad 26&60 Superdex 75 prep grade) (GE Healthcare, Chicago, USA) in NMR buffer (50 mM Tris, 200 mM NaCl, pH 8.0) and the monomeric protein was collected.

### Optimization of AMUPol and glycerol concentration for DNP-enhanced solid-state NMR experiments

To improve the enhancement factor *ε* and obtain the maximum signal intensity, different glycerol cushions and AMUPol were tested. We used 0–40% (v/v) glycerol with 15 mM AMUPol and 40% (v/v) glycerol cushion with 7.5 mM AMUPol. In each case, a 200 µl sample with a concentration of 11 µM (2.2 nmol) was loaded on a 500 µl glycerol cushion with the abovementioned concentrations of AMUPol and glycerol (Supplementary Table 3 and Fig. 10b). The sample was sedimented at 140,992.2 × *g* (28,000 r.p.m., Beckman Coulter, SW28) at 4 °C for 15 h using a centrifugation device as previously described^51^.

### DNP-enhanced solid-state NMR experiments

A 200 µl (10–17 µM, 2–3.4 nmol) sample was loaded on a 500 µl cushion of Tico buffer in D8-^13^C depleted-glycerol:D_2_O:H_2_O (42:45.3:14.5) (v/v) with a total AMUPol concentration of 15 mM (final volume 700 µl). The sample was sedimented at 140,992.2 × *g*, 4 °C, 15 h (28,000 r.p.m., Beckman Coulter, SW28) using a centrifugation device as previously described^51^. All DNP-enhanced solid-state NMR experiments were carried out on a Bruker Avance spectrometer operating at 9.4 T (400 MHz as ^1^H Larmor frequency). Due to a large molecular weight of the RNCs, only a few (2–3.4 nmol) can be packed into a rotor, which is two orders in magnitude lower than required for conventional solid-state NMR. Therefore, DNP enhancement is indispensable to increase signal to noise for ribosome samples that cannot be concentrated above 30 µM (Supplementary Fig. 10c). The spectrometer was equipped with a customized Bruker low-temperature HCN triple resonance probehead. The high-power microwave was generated from a CPI gyrotron (Palo Alto, USA) operating at 9.4 T and was directed to the sample position in the solid-state NMR probehead through corrugated waveguides. The effective microwave power emitted into the magic angle spinning (MAS) stator was about 12 W. In all experiments, MAS was stabilized at 8 kHz, sample temperature was kept at about 110 K, and an interscan delay of 3 s was used. ^1^H-^13^C CP 1D and ^13^C-^13^C DQ–SQ spectra were acquired with parameters similar to those previously reported^52^. Briefly, for the CP step, a ramped (80–100%) ^1^H RF field at 52 kHz as maximum power and a ^13^C RF pulse at 48.5 kHz were applied during 0.8 ms contact time. In the ^13^C-^13^C DQ–SQ experiments, the DQ coherence was excited and reconverted back to the SQ coherence by a train of POST-C7 recoupling pulses of 0.5 ms total length with field strength at 56 kHz. The DQ evolution period was rotor “synchronized”, permitting a large spectral window of the indirect dimension. During the ^13^C detection period, SPINAL64 ^1^H decoupling pulses with 100 kHz field strength were applied. The ^13^C-^13^C DQ–SQ 2D spectra were acquired with 60 and 2432 points on F1 (DQ, 556 ppm) and F2 (SQ, 296 ppm) dimension, respectively, and were processed with a 1024 × 8192 (F1 × F2) matrix. The Qsine (ssb = 2) and Gaussian (lb = −50 Hz, GB = 0.04) window functions were used for processing the indirect and direct ^13^C dimensions, respectively.

The ^1^H-^15^N CP experiment used for optimizing DNP sample preparations was acquired with 512 points and a spectral window of 600 ppm. During the 400 µs CP contact time, a ramped (80–100%) ^1^H RF field at 62 kHz as maximum power and a ^15^N RF pulse at 47 kHz were applied. In the ^15^N-^13^C TEDOR 2D experiment, the recoupling time was set to 0.5 ms (4 rotor periods). In the ^15^N-^13^C DCP 2D experiment, the ^15^N-^13^C magnetization transfer was realized by applying a ramped (90–100%) ^13^C RF pulse with 28 kHz field strength and a ^15^N RF pulse with 40 kHz field strength during the 5.6 ms contact time. The ^15^N-^13^C TEDOR spectrum was acquired with 192 and 1024 points on F1 (197 ppm) and F2 (296 ppm) dimension, respectively, and were processed with a 1024 × 4096 (F1 × F2) matrix. The ^15^N-^13^C DCP spectrum was acquired with 192 and 2048 points on F1 (197 ppm) and F2 (296 ppm) dimensions, respectively, and were processed with a 1024 × 4096 (F1 × F2) matrix. The Qsine (ssb = 2) and Gaussian (lb = −20 Hz, GB = 0.05) window functions were used for processing the indirect (^15^N) and direct (^13^C) dimensions. respectively. All ^13^C chemical shifts were referenced to sodium trimethylsilylpropanesulfonate (DSS) in water indirectly using an alanine powder sample. All ^15^N chemical shifts were referenced indirectly to liquid ammonia. Under our optimized sample conditions (Supplementary Table 3 and Fig. 10b), a sensitivity enhancement of approximately 150 was obtained at 100 K, which is eight times higher than that previously recorded for a ribosome sample^51–53^ and is comparable to that of other homogeneous protein preparations^52^.

### Solution-state NMR experiments

The full-length GBC was concentrated to around 800 µM in GBC NMR buffer (50 mM Tris, 200 mM NaCl, 10% D_2_O, 0.1% DSS, pH 8.0). Backbone assignment of GBC was done by performing ^1^H-^15^N BEST-TROSY and BEST-TROSY versions of the standard triple resonance experiments (HNCACB, HN(CO)CACB, HNCO, CC(CO)NH) on a Bruker spectrometer (AV800) equipped with a TCI cryo probe. All spectra were recorded at 298 K. The 3D spectra were recorded with an exponential weighting of sampling scheme, processed using the software Topspin 3.5 (Bruker BioSpin, Rheinstetten, Germany) and analyzed using the software Cara 1.8.4.2 (ref. ^54^) or Topspin 3.5–4.08 (Bruker BioSpin).

### Analysis of solid-state NMR spectra of RNCs

All chemical shift assignments on ^13^C-^13^C DQ–SQ spectra were carried out on Topspin 3.5 and Sparky^55^. Because C_β_ chemical shift is the mostly indicative parameter for the Cys chemical states and C_α_ chemical shift is sensitive to Cys backbone conformation, we focused primarily on assigning the signals in the C_α_–C_β_ region in our spectra. We first assigned those resolved C_β_ signals in the C_α_/C_β_ (DQ)-C_β_ (SQ) region and the resolved C_α_ signals in the C_α_/C_β_ (DQ)-C_α_ (SQ) and C_α_/C′(DQ)-C_α_ (SQ) region. Based on these assignments, the correlated C_α_ and C_β_ signals were then extracted from the spectra. Integration of the C_β_ region for the determination of the relative integration ratio was carried out with Topspin 3.5 using the a+-mode. For the calculation of the integration error, the chemical shift region was varied slightly by +2 ppm for the oxidized and by −2 ppm reduced region in the SQ chemical shift dimension. The integration region of the oxidized signals was between 49.68 and 35 ppm (SQ), of the overlapping region between 35 and 33 ppm (SQ), and for the reduced region signals between 32 and 23.12 ppm (SQ). The DQ chemical shift dimension for the integration regions was between 121 and 69 ppm. Four different integration areas were used and the mean and standard error was calculated using R^56^.

### Secondary structure analysis of cysteine residues within RNCs

The C_α_ and C_β_ chemical shifts of the cysteine residues within the ^13^C-^13^C DQ–SQ of the RNCs were extracted and saved as a.csv file. The files were loaded into R^56^. Chemical shift data of cysteine residues within different structural elements derived from the BMRB^57^ were taken from Wang et al.^24^. The C_α_ chemical shifts were plotted as a function of the C_β_ chemical shifts. An ellipse containing 90% of the data was drawn around each structural element. The individual C_α_ and C_β_ of the isolated GBC and the RNCs were plotted within this data set. C_β_ chemical shifts above 35 ppm correspond to oxidized cysteine resides, C_β_ chemical shifts between 35 and 32 ppm are in the overlapping region, and can be either oxidized or reduced, and Cβ chemical shifts below 32 ppm correspond to reduced cysteine residues.

### Chemical shift determination of GSNO, GSH, and GSSG

Commercially available GSNO (Sigma-Aldrich, St. Louis, USA), GSH (Carl Roth, Karlsruhe, Germany), and GSSG (Carl Roth, Karlsruhe, Germany) were resolved in GBC NMR buffer (50 mM Tris, 200 mM NaCl, 10% D_2_O, pH 8.0) with 1 mM trimethylsilylpropanoic acid (TSP) to a concentration of approximately 50 mM. ^13^C 1D NMR spectrum was acquired with 2048 number of scans and 65,536 points at 298 K on a Bruker spectrometer (AV500HD). The spectrum was processed with Topspin 4.08 and the chemical shifts were referenced to TSP. C_α_ and C_β_ chemical shifts of the cysteine residue within the different GSH variants were determined.

### Preparation of cryo-EM sample and image processing

A 3.5 µl aliquot of an approximately 50 nM sample of U32SecM in Tico buffer was applied to a glow-discharged (20 s) Quantifoil R1/2 holey carbon grid (Cu 300 mesh, Quantifoil Mirco Tools GmbH, Jena, Germany) coated with an approx. 5-nm-thick carbon film^58^. Grids were plunge-frozen in liquid ethane using a Vitrobot Mark IV (FEI, Hillsboro, OR) with a blotting time of 8–9 s, a blotting force of −1 to −5, at 4 °C and 100% humidity. Dose-fractionated movie stacks (36 frames, 0.2 s each) were collected at a nominal magnification of ×130,000 (1.05 Å pixel size) with a 300 kV Titan Krios (FEI) microscope in nanoprobe EFTEM mode, equipped with a K2 Summit detector (Gatan, Pleasanton, CA) and a GIF Quantum s.e. post-column energy filter in zero loss peak mode. The defocus values varied from −0.7 to −3.5 µm. Data were recorded using SerialEM^59^ in a semi-automated way. Image processing was done in Relion 3.1 (refs. ^60,61^) using Shawn Zheng’s MotionCor2 (ref. ^62^) for beam-induced motion correction and Niko Grigorieff’s CTFFIND4.1 (ref. ^63^) to estimate the contrast transfer function parameters. A total of 436,999 particles were automatically picked using 2D class references from manually picked particles. Bad particles were removed using reference-free 2D classification and the remaining 326,344 particles were further 3D classified into six classes with an angular sampling rate of 7.5°. The reference map for the 3D classification was generated from the 70S Ribosome-SecM (PDB 3JBV) model in USCF Chimera^64^ and low-pass filtered to 60 Å in Relion. Classes with low density for the 30S subunit were removed and 266,000 particles were used for further 3D refinement using an initial angular sampling of 7.5° and local angular searches of 1.8°. The refined structure was further post-processed using a soft mask generated by extending the binarized 15 Å low-pass filtered map and addition of a cosine-shaped edge yielding a structure of 3.52 Å. *B*-factor for sharpening of the map was automatically determined by Relion (−102.4).

We used the CTF refinement functionality of Relion 3.1 to estimate per-particle defocus values with a search range of 200 nm and without beamtilt estimation. CTF refinement was followed by Bayesian polishing using all frames of the movie stacks and parameters that were determined during a training run using 10,000 particles. CTF refinement and Bayesian polishing improved the resolution of the reconstruction to 2.98 Å. In order to resolve the structural heterogeneity of the reconstruction, we 3D classified the particles into four classes using a mask comprising the large subunit and all tRNA states, plus a regularization parameter of 4, and without performing image alignment. Besides one very low-populated class with potential junk particles, we identified two classes showing clear tRNA density in the P-Site (196,000 particles and 13,000 particles) and one class that showed clear tRNA density in the P-site but slightly shifted compared to the other classes (55,000 particles). We refined the highly populated class with tRNA density in the P-site and the class with shifted tRNA density in the P-site independently. Refinement of the high populated class converged at 2.83 Å resolution, showing the tRNA located in the P-site, whereas the other class converged at 3.19 Å resolution, showing the tRNA density in P/E and in the A/P* site. To further improve the resolution of the 2.83 Å reconstruction we estimated the magnification anisotropy, refined the defocus per-particle and the astigmatism per-micrograph followed by estimation of beamtilt and trefoil with the CTF refinement of Relion 3.1. The final 3D refinement and reconstruction that corrected these effects converged at 2.58 Å resolution.

### Model building

As an initial reference model for the SecM-stalled RNC, the cryo-EM structure of the 70S *E. coli* ribosome (PDB 3JBU) was chosen. The model was fitted into the cryo-EM electron density map of the *E. coli* BL21 (DE3) ribosome using the *Fit in Map* tool of UCSF Chimera^64^. To focus in detail on the structural analysis of the 50S ribosomal subunit, the 30S subunit could be removed from the model. Therefore, structural dynamics between both subunits were not analyzed. For an initial real-space refinement of all 50S subunit RNA, tRNA, and protein components the *Rigid Body Fit Zone* tool of COOT^65^ was used. Since the density of the NC was rather faint in the *B*-factor corrected sharpened map, we mutated the ten N-terminal residues of the NC sequence to the GBC sequence using COOT. Afterwards, the model was refined using the *Real-space refine (cryo-EM)* tool of PHENIX^66^. Model validation and calculation of FSC curves were carried out using the PHENIX *Comprehensive validation (cryo-EM)* tool. Furthermore, the validated model was manually analyzed using COOT. Ramachandran outliers, unusual rotamers, and potential clashes were inspected and adjusted if necessary. In addition, protein and RNA sequence deviations of *E. coli* K12 and *E. coli* BL21 (DE3) ribosomal 50S subunit were corrected manually. Subsequently, several cycles of refinement and validation were carried out using COOT and PHENIX. The cryo-EM electron density maps of GBC-SecM-RNC (50S subunit) have been deposited in the Electron Microscopy Data Bank under accession code 4531. Their associated atomic models have been deposited in the Protein Data Bank under accession code 6QDW.

To build the NC, we than used the unsharpened map, which showed the NC density much more clear. We rebuilt the previously refined NC (PDB: 6QDW) and added GBC residues until the density became too faint for proper model building. Model validation and calculation of FSC curves were carried out using the PHENIX *Comprehensive validation (cryo-EM)* tool. Furthermore, the validated model was manually analyzed using COOT. Ramachandran outliers, unusual rotamers, and potential clashes were inspected and adjusted if necessary. Subsequently, several cycles of refinement and validation were carried out using COOT and PHENIX. The cryo-EM electron density maps of GBC-SecM-RNC (50S subunit) have been deposited in the Electron Microscopy Data Bank under accession code 10891. Their associated atomic models have been deposited in the Protein Data Bank under accession code 6YS3. Statistical parameters are given in Supplementary Table 4.

### Simulation of conformational sampling of newly synthesized polypeptide in the ribosomal exit tunnel

The degrees of freedom available to the nascent polypeptide chain were simulated using an adapted version of the statistical coil sampling engine flexible-meccano^26,67^. Backbone degrees of freedom were defined by amino acid-specific potentials derived from loop regions extracted from a database of high-resolution crystal structures^68^. Steric clashes were taken into consideration using a residue-specific hard sphere model^69^. Conformational sampling of U32SecM was initiated at position Ser151 of SecM and the polypeptide was built in the direction from C to N using a self-avoiding algorithm that randomly samples the backbone potentials. Only self-avoiding conformations and conformers that avoid steric clash with all heavy atoms from the coordinates of the tunnel (determined from cryo-EM; see above) are retained for further analysis. The calculation was repeated for 20,000 conformations.

The potential of cysteine amino acids (Cys15, Cys18, Cys22, and Cys32) to form disulfide bridges was assessed by measuring the distances between cysteine C_β_ atoms within each conformation, with a threshold of 4.5 Å. The same calculations were repeated in the presence and absence of the ribosomal exit tunnel to assess the relative importance of internal degrees of freedom and steric interaction with amino acids forming the tunnel wall.

### LC-MS/MS measurements of RNCs

Purified protein samples were initially diluted in 50 mM ammonium bicarbonate buffer and treated with 10 µg RNAse A (Qiagen, Hilden, Germany) for 10 min, followed by the alkylation of free cysteine residues with iodoacetamide (Thermo Fisher Scientific, Waltham, USA) for 20 min in the dark. Proteins were then digested in solution by the addition of 1 µg trypsin (Serva, Heidelberg, Germany) and incubated for 3.5 h at 37 °C.

Proteolytic digests were finally desalted using C18 ZipTips (Merck, Darmstadt, Germany) according to the manufacturer’s instructions, dried in a vacuum concentrator (Eppendorf, Hamburg, Germany), and reconstituted in water/acetonitrile 95/5/0.1 (v/v) with 0.1% formic acid.

Subsequent LC-MS/MS analyses were carried out either on a nanoElute coupled to an Impact II mass spectrometer (Bruker Daltonics, Billerica, USA) or an Ultimate3000 RSLCnano coupled to a Fusion Lumos mass spectrometer (Thermo Fisher Scientific, Waltham, USA) with the parameters given in Supplementary Table 5.

Mass spectra were acquired over the mass range 150–2200 *m*/*z* (Impact II)/350–1600 *m*/*z* (Fusion Lumos) and sequence information was acquired by a computer-controlled, dynamic method with a fixed cycle time of 3 s and intensity-dependent acquisition speed for MS/MS-spectra of the most abundant candidate ions.

The resulting files were exported to the open MZML format (Impact II) and recalibrated, and database-, PTM- and crosslink searches were performed using the Metamorpheus search engine against a combined database containing the *Escherichia coli* proteome (obtained from Uniprot, 11/2019), the respective SecM constructs and common contaminants.

The search parameters used were as follows: <20 ppm mass tolerance for precursor and fragment ions, fully tryptic cleavages with up to two missed cleavages and heavy labeled cysteine, carbamidomethylation (C), gluthathione (C), oxidation (M) as variable modifications. Further modifications were analyzed with GPTMD (common artefacts and biological modifications) and disulfide crosslinks were searched for light and heavy labeled cysteines.

### Figure preparation

All figures were prepared using Pymol 4.6 (Schrödinger, LLC.), USCF Chimera 1.14 (ref. ^64^) or USCF ChimeraX 1.0 (ref. ^71^).

### Reporting summary

Further information on research design is available in the Nature Research Reporting Summary linked to this article.

## Supplementary information

Supplementary Information

Reporting Summary

Soure Data

## Data Availability

The data that support this work are available from the corresponding authors upon reasonable request. The mass spectrometry proteomics data have been deposited to the ProteomeXchange Consortium via the PRIDE^70^ partner repository with the data set identifier PXD021574. PDB entries 3JBV and 3JBU (Electron Microscopy Data Bank under accession code of EMD-6483 and EMD-6486) were used for 3D classification and model building. Structural models generated in this study have been deposited in the Protein Data Bank with accession codes [6QDW] and [6YS3] and in the Electron Microscopy Data Bank with accession codes EMD-4531 and EMD-10891. The data set from Wang et al.^24^ was used to draw the ellipsis in the Cα-Cβ plots. Each ellipse contains 90% of the corresponding secondary structure. Source data are provided with this paper.
