# Supplementary material for: Cysteine oxidation and disulfide formation in the ribosomal exit tunnel
Source: Nat Commun. 2020 Nov 4;11:5569. doi: 10.1038/s41467-020-19372-x (PMC7642426; doi:10.1038/s41467-020-19372-x)
Supplement: Supplementary file 1 — Supplementary Information [file 41467_2020_19372_MOESM1_ESM.pdf]

Supplementary Information

Supplementary Table 1: Identified loop-linked disulfides and cysteine modifications with glutathione or nitric oxide in NCs. Free, reduced cysteines in RNCs measured by LC/MS-MS were alkylated with iodacetamid (Methods). Disulfide bond of U32SecM (cryo-EM) was determined by cryo-EM. Full-length GBC was measured by liquid-state NMR and oxidation ratio was determined by peak integration.

Construct	identified disulfide bond	Trypsin cleavage site between peptides	% of oxidation	Cysteine modification
U32SecM C18	-		19	S-nitrosylation (Cys18)<br>S-glutathionylation (Cys18)<br>carbamidomethyl (Cys18)
U32SecM C15 C18	.		19	.
U32SecM C18 C22	-		16	carbamidomethyl (Cys18, Cys22)
U32SecM	Cys15-Cys32<br>Cys22-Cys32	yes<br>yes	25	carbamidomethyl (Cys15, Cys18, Cys22, Cys32)
U32SecM (cryo-EM)	Cys22-Cys32	yes	63	

U43SecM	Cys32-Cys41	no	38	S-nitrosylation (Cys22, Cys41)<br>carbamidomethyl (Cys15, Cys22, Cys32, Cys41)
U78SecM	Cys32-Cys41	no	34	S-nitrosylation (Cys41)<br>carbamidomethyl (Cys15, Cys22, Cys32, Cys41, Cys78)
Full-length GBC	-		Cys22 7.4% oxidized<br>Cys78 9.3% oxidized	
Full-length GBC Cu(II) oxidized	Cys22-Cys78		Cys22 39.2% oxidized<br>Cys78 53.7% oxidized	

Supplementary Table 2: Composition of M9 minimal medium.

M9 minimal medium	5000x Trace elements	2000x Vitamin mix
42 mM Na_2HPO_4	50 mM $FeCl_3$	100 mM Thiamine (vitamin B_1)
22 mM KH_2HPO_4	20 mM $CaCl_2$	100 mM Biotine (vitamin H)
8.5 mM NaCl	10 mM $MnCl_2$	100 mM Niacin (vitamin B_3)
0.2 mM $CaCl_2$	10 mM $ZnSO_4$	10 mM Cobalamin (vitamin B_{12})
2 mM $MgSO_4$	2 mM $CoCl_2$	
1x Vitamin mix	2 mM $CuCl_2$	
1x Trace elements	2 mM $NiSO_4$	
1 g/L $^{15}NH_4Cl$	2 mM Na_2MoO_4	
2 g/L ^{13}C-glucose	2 mM Na_2SeO_3	
100 µg/ml ampicillin	2 mM H_3BO_3	

Supplementary Table 3: Optimization of glycerol cushion. Glycerol concentration in % (v/v), AMUPoL (mM), ^{1}H enhancement factor ε, relative signal intensity with and without microwave (MW).

Sample	Glycerol % (v/v)	AMUPol (mM)	^{1}H Enhancement factor ε	Relative NMR signal intensity (MW off)	Relative NMR signal intensity (MW on)
I	40	7.5	169	0.20	0.65
II	40	15	130	0.23	0.59
III	30	15	131	0.39	1.00
IV	20	15	41	0.97	0.78
V	10	15	67	0.65	0.85
VI	0	15	35	1.00	0.68

Supplementary Table 4: Experimental conditions of data collection, refinement and model statistics of 6QDW and 6YSE.

**Data Collection**	6QDW	6YS3
Particles	196254	196254
Pixel size (Å)	1.05	1.05
Defocus range (µm)	0.7-3.5	0.7-3.5
Voltage (kV)	300	300
Electron dose (e^-/Å^2)	60	60

Model Composition		
Protein residues	3127	3136
RNA bases	3091	3091
**Refinement**		
Resolution (Å, 0.143 FSC)	2.83	2.58
Map sharpening B-factor	-44.45	-34.52
**Validation**		
RMSZ, bonds	1.30	1.24
RMSZ, angles	1.15	1.11
Rotamer outliers (%)	1.0	0.8
Ramachandran outliers (%)	0.1	0.0
Ramachandran favoured (%)	93	93
Correct sugar pucker (%)	100	100
Good backbone conformations (%)	73	73
**Scores**		
MolProbity	1.88	1.81
Clash score, all atoms	6.27	6.4

Supplementary Table 5: Systems, columns and experimental conditions for mass spectrometry performance on RNCs.

System	nanoElute / Impact II	Ultimate3000 / Fusion Lumos
**Columns**	Triart C18, 5*0.3 mm, 1.9 µm (YMC)	Pepmap C18, 20*0.075 mm, 3 µm (Thermo Fisher)
	Reprosil C18, 16*0.0075 mm, 1.9 µm (Pepsep)	Pepmap C18, 500*0.075 mm, >2 µm (Thermo Fisher)
**Mobile phase**	A: Water + 0.1% FA	A: Water + 0.1% FA
	B: Acetonitrile + 0.1% FA	B: 80% Acetonitrile + 0.1% FA
**Gradient**	Linear 2-35% B, 90 min	Linear 4-48% B, 178 min
**Flow rate**	300 nl/min	
**Temperature**	55°C	

Supplementary Table 6: Interactions between the NC and the ribosomal proteins L4, L22 and 23S rRNA. NC (chain z), ribosomal protein L4, L22 (chain e and chain s) and 23S rRNA (chain b).

NC residues	Interacting residue, ribonucleotide		
**Atom, Residue**	**Atom, Residue (three letter code) or ribonucleotide (one letter code)**	**Chain**	**Type of interaction**
O (CO), Trp155	H (NH_2), Arg61	e	Hydrogen bonding

Trp155	A753	b	π-π stacking
H (NH), Arg31	OP1 (PO_4), C1259	b	Hydrogen bonding
H (NH_2), Arg31	OP2 (PO_4), U1260	b	Hydrogen bonding
H (OH), Ser30	OP1 (PO_4), U1260; OP2 (PO_4), U1260	b	Hydrogen bonding
H2 (NH_2), Asn24	OP1 (PO_4), C461; O3’, C462	b	Hydrogen bonding
S, Cys22	S, Cys32	z	Disulfide bond
O (CO), Ser19	H (NH_2), Arg84	s	Hydrogen bonding
O (OH), Ser20	H (NH_2), Arg95	s	Hydrogen bonding
H (OH), Tyr16	O (PO_4), A508	b	Hydrogen bonding

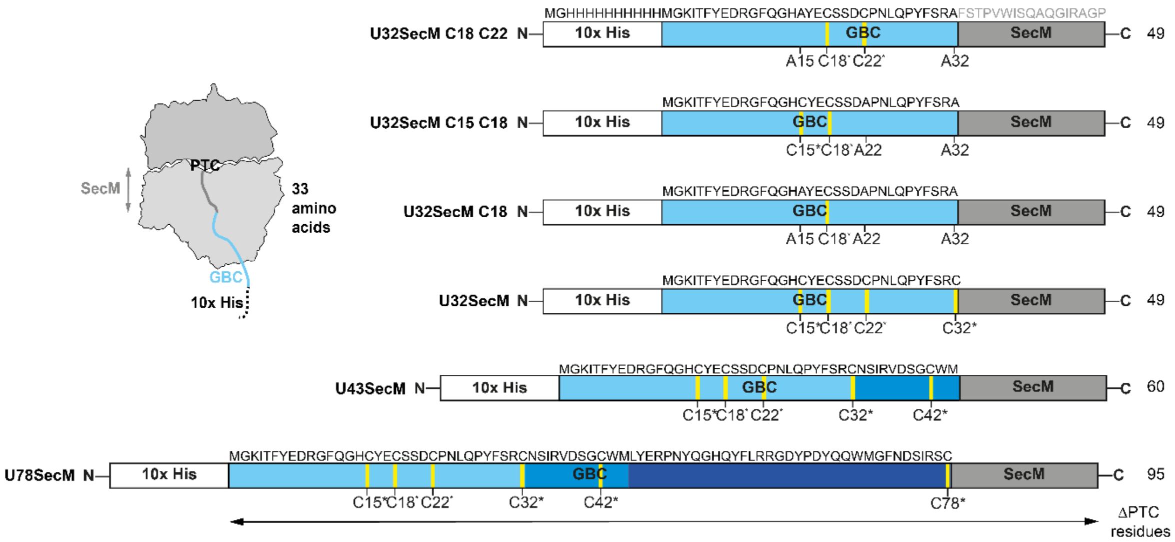

Supplementary Figure 1: **RNC GBC constructs with variable polypeptide chain lengths.** Different lengths of the GBC gene were fused with an N-terminal 10x histidine purification tag (white) and the 17-amino-acid SecM stalling sequence (dark grey). Numbering excludes the N-terminal methionine of GBC to be consistent with the literature residues 1-32 (light blue), 33-43 (blue) and 44-78 (dark blue). ^{13}C-labeled Cys residues are marked with an asterisk (*) and highlighted in yellow.

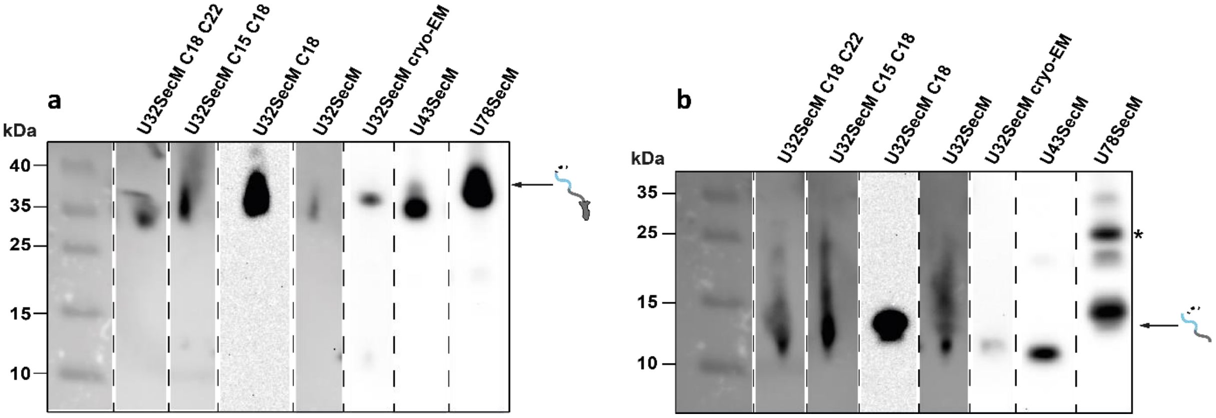

Supplementary Figure 2: **Western blot of RNC GBC constructs with variable polypeptide chain lengths. a)** Western blot analysis of purified ribosome-bound RNC. **b)** Western blot analysis of RNCs after treatment with RNaseA. NC was detected with a poly-histidine antibody (Merck, Darmstadt, Germany). The western-blots were carried out on different gels after protein purification and were aligned to the protein marker. U78SecM showed dimer formation after release from the ribosome even under reducing conditions (*). Variations of the background are a result of an update of the imaging software. For each of those constructs different isotopically enriched samples were prepared and had a consistent pattern in the western blot.

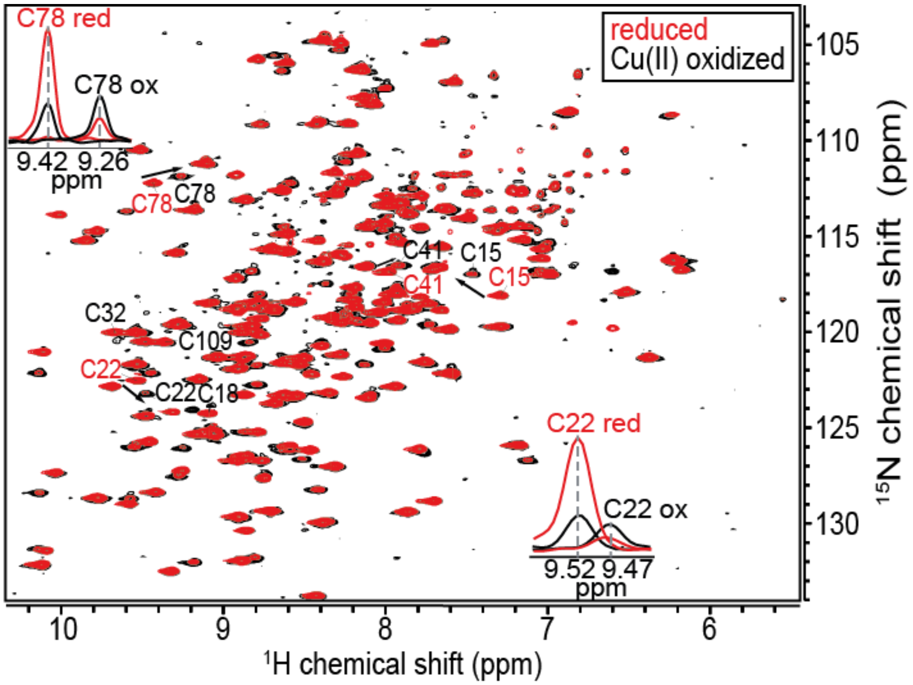

Supplementary Figure 3: **2D backbone ^{1}H-^{15}N-HSQC NMR spectrum of GBC full-length reduced (red) and after oxidation with Cu(II) (black).** Positions of the cysteine residues are labeled in red and black accordingly. Cysteine residues not changing position after oxidation (Cys18, Cys109) or that could not be unambiguously identified due to overlapping signals (Cys32) were only labeled once. 1D traces of Cys22 and Cys78 peaks are shown in the panel. Cu-(II) oxidation led to reduction in the protein yield and a decrease in the signal/noise ratio for the oxidized protein.

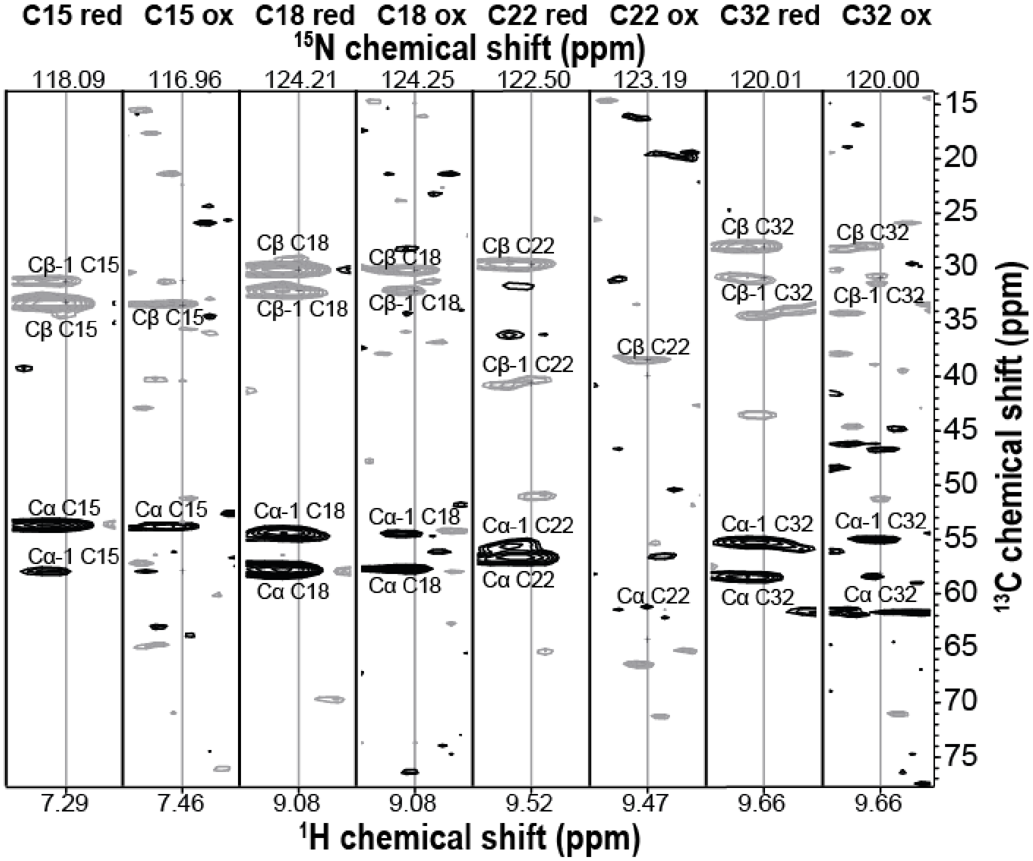
C15 red
C15 ox
C18 red
C18 ox
C22 red
C22 ox
C32 red
C32 ox
15N chemical shift (ppm)
118.09
116.96
124.21
124.25
122.50
123.19
120.01
120.00
Cβ-1 C15
Cβ C15
Cα C15
Cα-1 C15
Cβ C15
Cα C15
Cβ C18
Cβ-1 C18
Cα-1 C18
Cα C18
Cβ C18
Cβ-1 C18
Cα-1 C18
Cα C18
Cβ C22
Cβ-1 C22
Cα-1 C22
Cα C22
Cβ C22
Cα C22
Cβ C32
Cβ-1 C32
Cα-1 C32
Cα C32
Cβ C32
Cβ-1 C32
Cα-1 C32
Cα C32
7.29
7.46
9.08
9.08
9.52
9.47
9.66
9.66
1H chemical shift (ppm)
13C chemical shift (ppm)
15
20
25
30
35
40
45
50
55
60
65
70
75

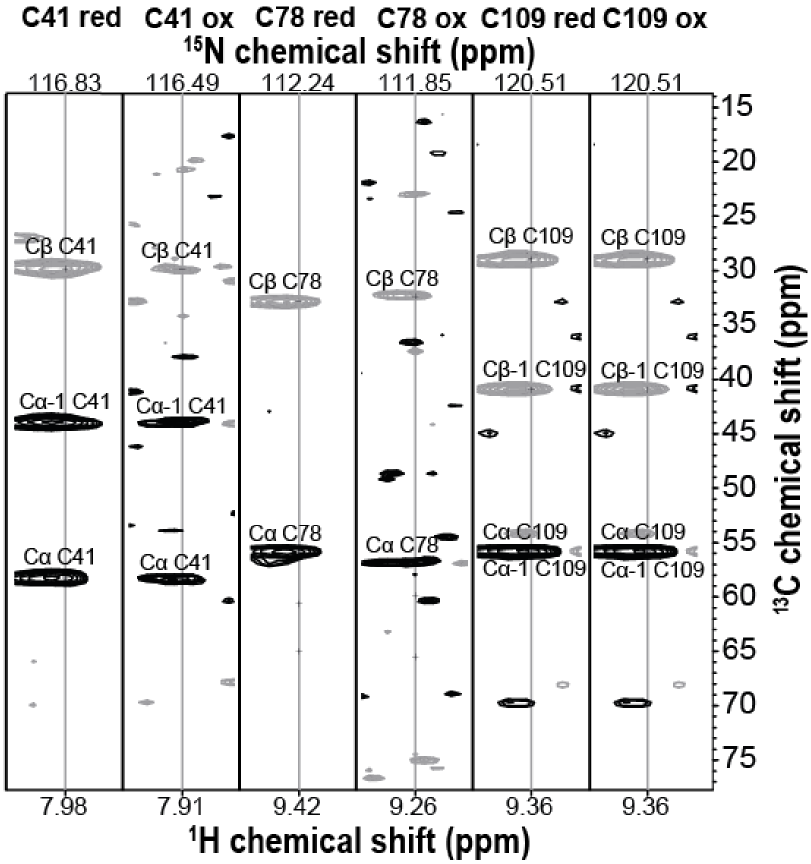
C41 red
C41 ox
C78 red
C78 ox
C109 red
C109 ox
15N chemical shift (ppm)
116.83
116.49
112.24
111.85
120.51
120.51
Cβ C41
Cα-1 C41
Cα C41
Cβ C41
Cα-1 C41
Cα C41
Cβ C78
Cα C78
Cβ C78
Cα C78
Cβ C109
Cβ-1 C109
Cα C109
Cα-1 C109
Cβ C109
Cβ-1 C109
Cα C109
Cα-1 C109
7.98
7.91
9.42
9.26
9.36
9.36
1H chemical shift (ppm)
13C chemical shift (ppm)
15
20
25
30
35
40
45
50
55
60
65
70
75

Supplementary Figure 4: ^{1}H-^{13}C strip plots from the BEST-TROSY HNCACB spectra of GBC full-length reduced and after oxidation with Cu(II). The C_α resonances are phased to yield positive (black) cross-peaks, whereas the C_β cross-peaks are negative (grey) The corresponding ^{15}N frequencies are at the top. After oxidation with Cu(II) and additional purification by size exclusion chromatography, approx. 30% of the protein yield was lost, leading to a decreased signal/noise ratio.

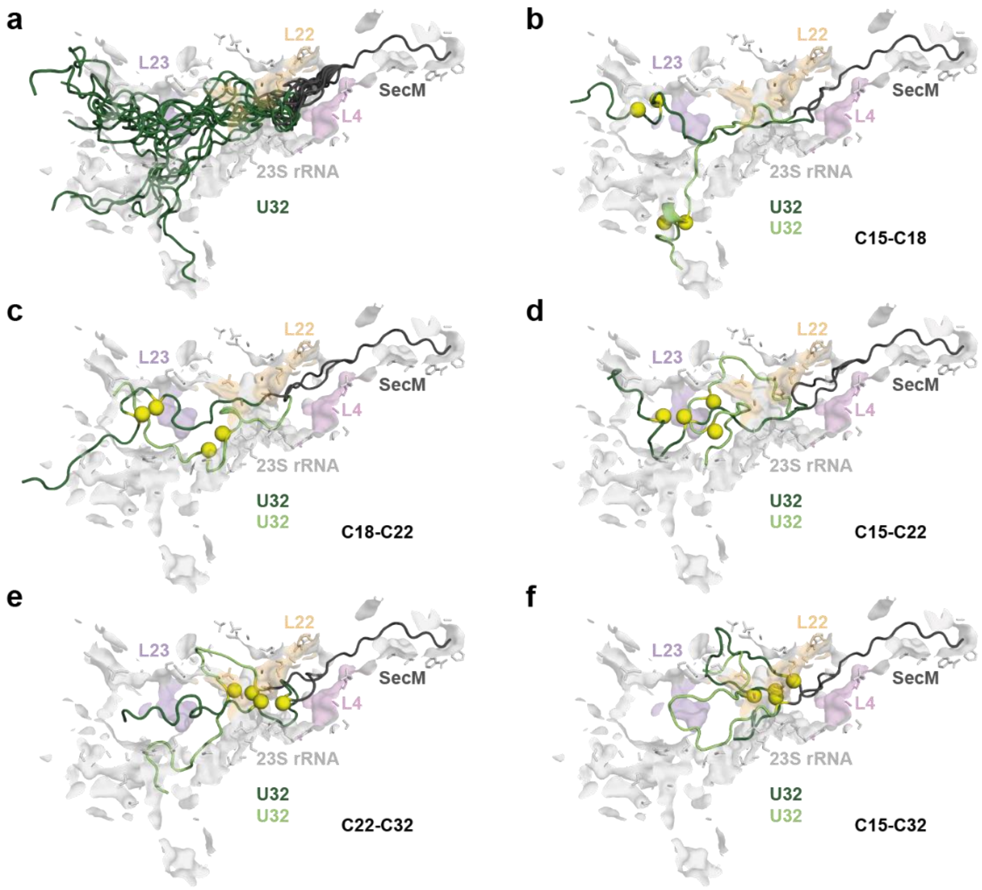

Supplementary Figure 5: Simulation of U32SecM within the ribosomal exit tunnel of *E. coli* using flexible-meccano. a) Ensemble of structures within the tunnel. U32SecM chains with Cys15-Cy18 **(b)**, Cys18-Cys22 **(c)**, Cys15-Cys22 **(d),** Cys22-Cys32 **(e)** and Cys15-Cys32 **(f)** in

close proximity for possible disulfide bridge. Cysteine residues are highlighted with a yellow ball. SecM in dark grey, U32 in green. Ribosomal proteins L4 (light pink), L22 (light orange) and L23 (light violet) and 23S rRNA (light grey) shape the ribosomal tunnel. The simulation included residues from Asp8 in GBC to Trp155 in SecM. The tunnel dimensions of PDB 6YSE were used.

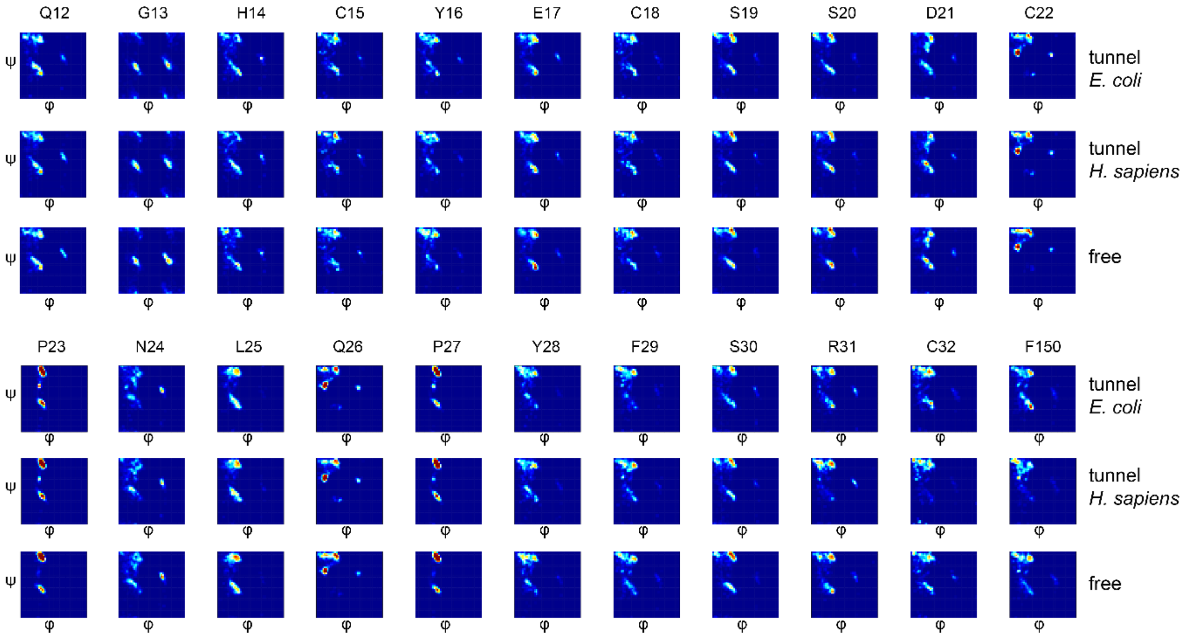

Supplementary Figure 6: Ramachandran plots of the simulated U32SecM NC in the ribosomal exit tunnel and free in solution. The tunnel dimensions of *E. coli* (PDB: 6YS3) and *H. sapiens* (PDB: 4UG0) were used for the simulation of the NC using flexible-meccano[26]. High population of the NC in red and low population in blue.

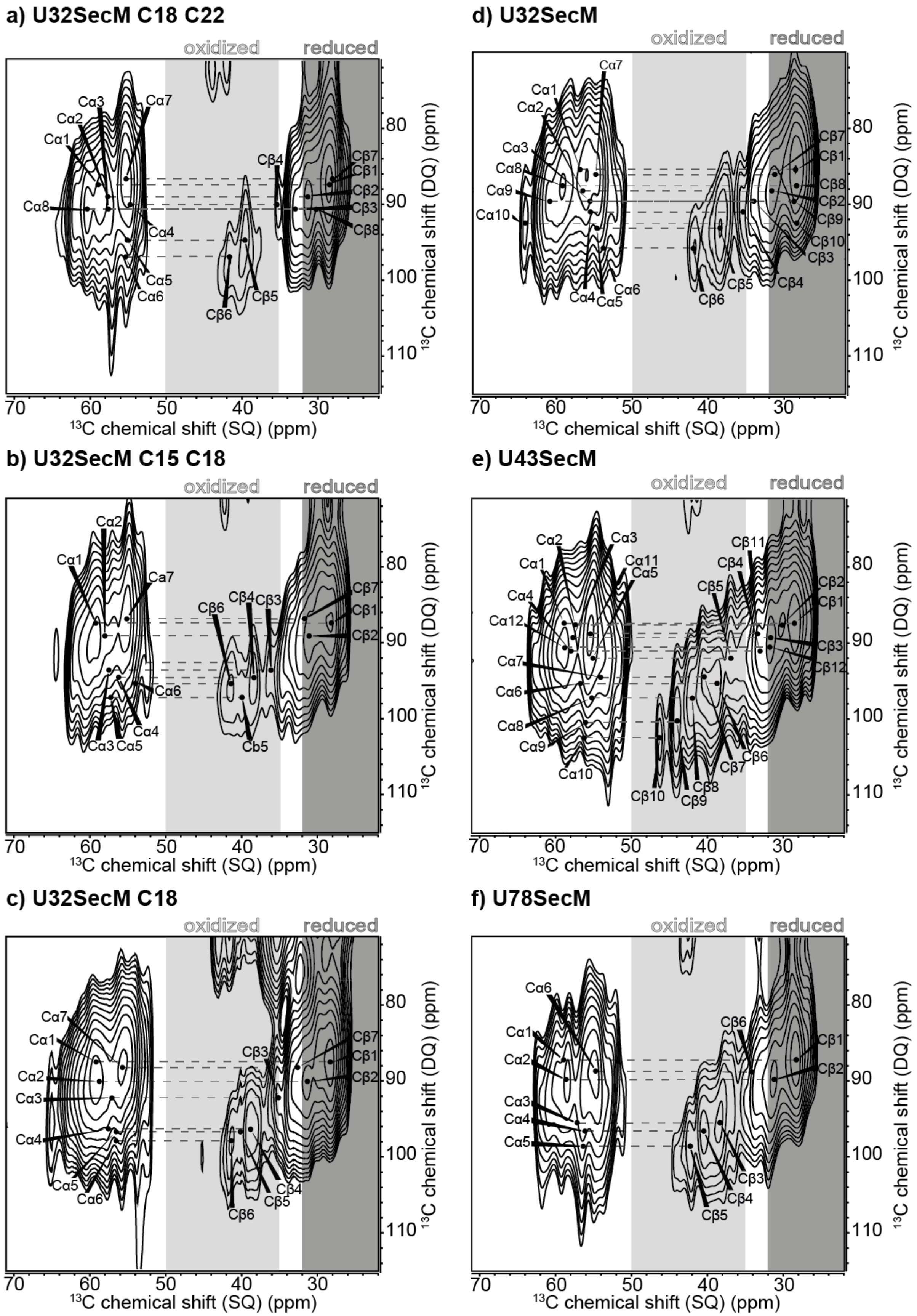

a) U32SecM C18 C22
b) U32SecM C15 C18
c) U32SecM C18
d) U32SecM
e) U43SecM
f) U78SecM
oxidized
reduced
^{13}C chemical shift (SQ) (ppm)
^{13}C chemical shift (DQ) (ppm)

Supplementary Figure 7: Cysteine C_α-C_β cross-peak regions of the ^{13}C DQ-SQ spectrum of ^{13}C, ^{15}N cysteine labeled RNCs. **a**) U32SecM C18 C22, **b)** U32SecM C15 C18, **c)** U32SecM C18, **d)** U32SecM, **e)** U43SecM and **f)** U78SecM (numbering scheme as given in Fig. 1). The cross-peak region for oxidized cysteine residues is highlighted in light grey, and for reduced cysteine residues in dark grey.

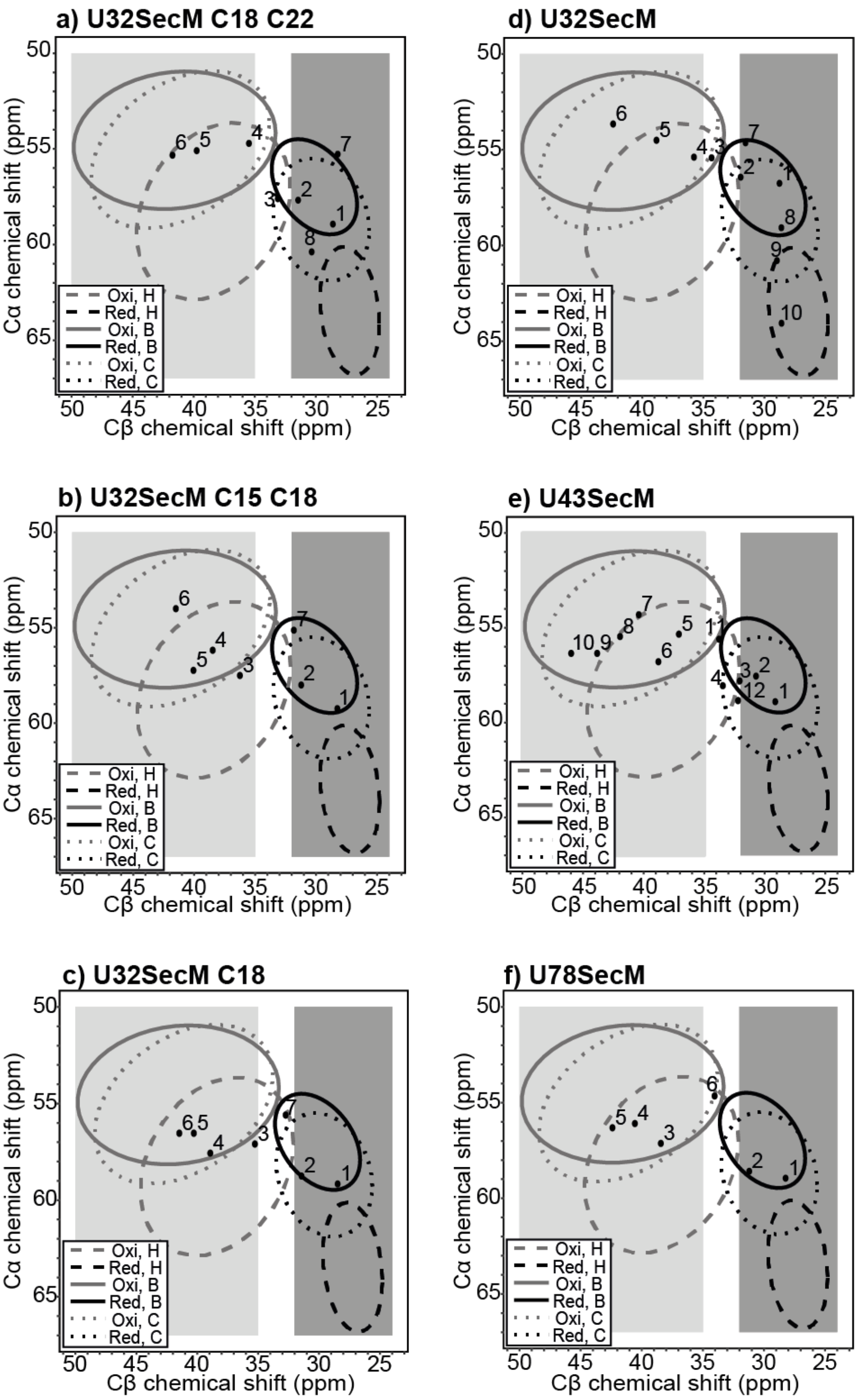

Supplementary Figure 8: Cα and Cβ chemical shifts from RNC constructs compared to values derived from a chemical shift database[24]. **a**) U32SecM C18 C22, **b)** U32SecM C15 C18, **c)** U32SecM C18, **d)** U32SecM, **e)** U43SecM and **f)** U78SecM (numbering scheme as given in Fig.

1). Ellipses show 90% of the corresponding chemical shifts. Secondary structures: H = helix, B = β-strand and C =coil. The cross-peak region for oxidized cysteine residues is highlighted in light grey, and for reduced cysteine in dark grey.

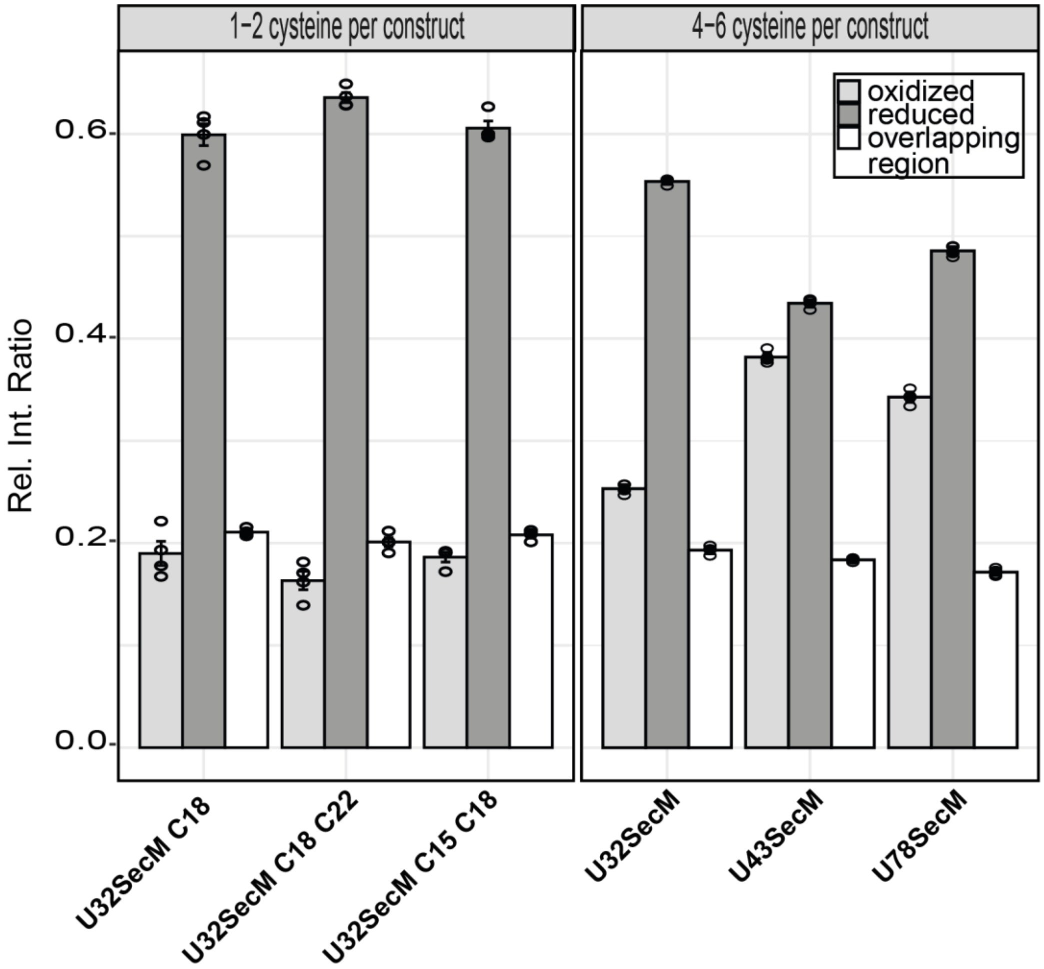

Supplementary Figure 9: Oxidation ratios of GBC RNCs. Relative integration ratio (Rel. Int. Ratio) of oxidized, reduced or overlapping regions, the last of which does not allow for differentiation, of all RNC spectra. Constructs contain 1-2 cysteine residues (left), 4-6 cysteine residues (right). Signal intensity of oxidized cysteine residues (>35 ppm), reduced cysteine residues (<32 ppm) and the overlapping region (35–32 ppm) was divided by the total signal intensity of Cβ chemical shifts. One sample per RNC construct was measured using solid-state NMR. Error bars represent the standard error of the mean and were calculated using four different integration regions (Methods). Each circle represents the Rel. Int. Ratio of one individual integration region.

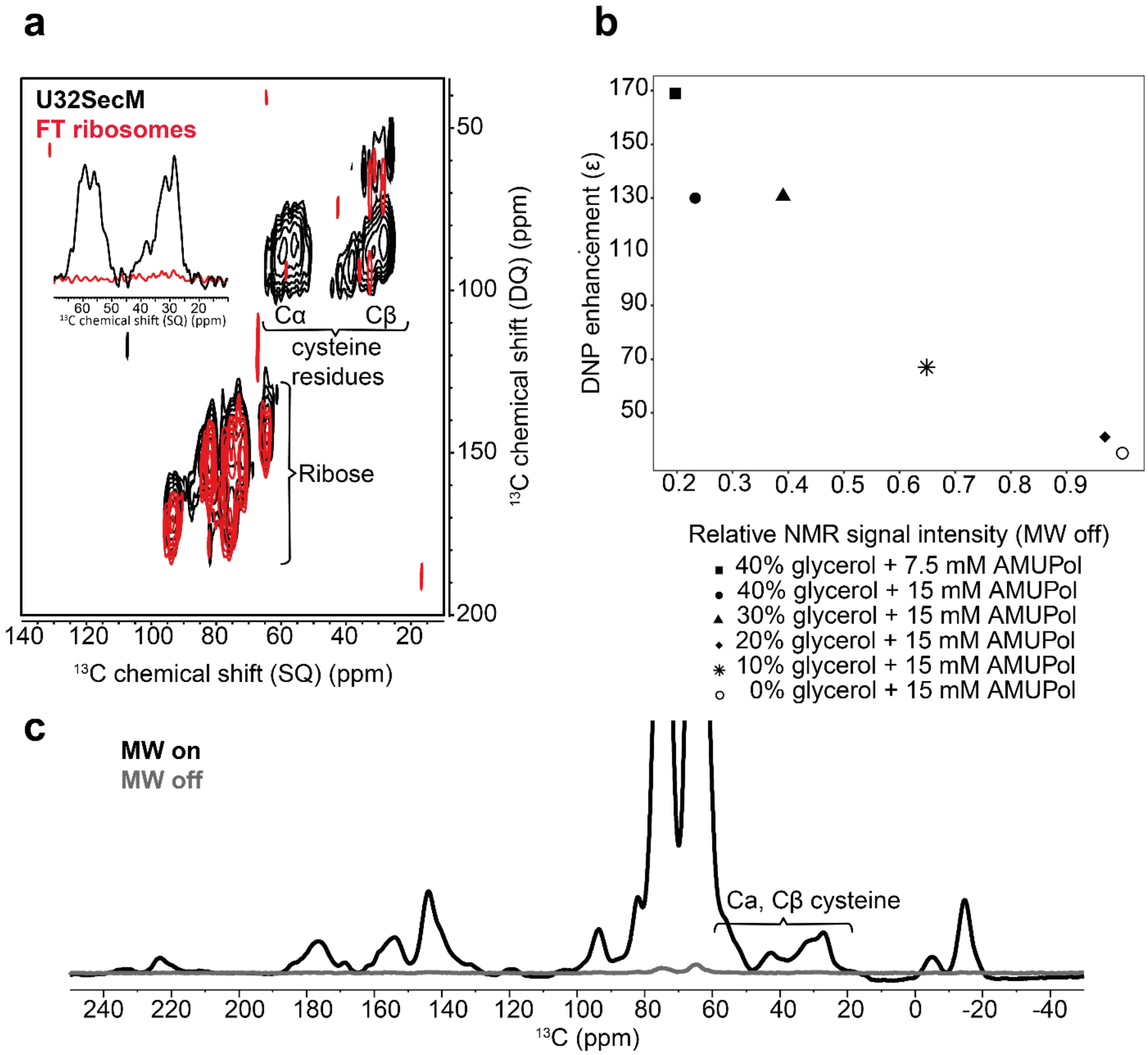

Supplementary Figure 10: Overlay of ^{13}C DQ-SQ solid-state NMR spectrum of flow-through ribosomes and U32SecM and DNP enhancement factor depending on glycerol conditions. a) ^{13}C POST-C7 double-quantum to single-quantum (DQ-SQ) spectra of the ^{13}C, ^{15}N labeled cysteine NC U32SecM (black) and flow-through (FT) ribosomes grown in the same media (red). No signals are observable within the cysteine Cα and Cβ region of the FT ribosomes. **b)** DNP enhancement factor of different glycerol cushion conditions plotted against relative NMR signal intensity with microwave (MW) turned off. **c)** ^{13}C CP 1D spectrum of ^{13}C, ^{15}N selectively cysteine labeled U78SecM with MW turned on and off. NS= 512, T=100K, MAS frequency = 8 kHz.

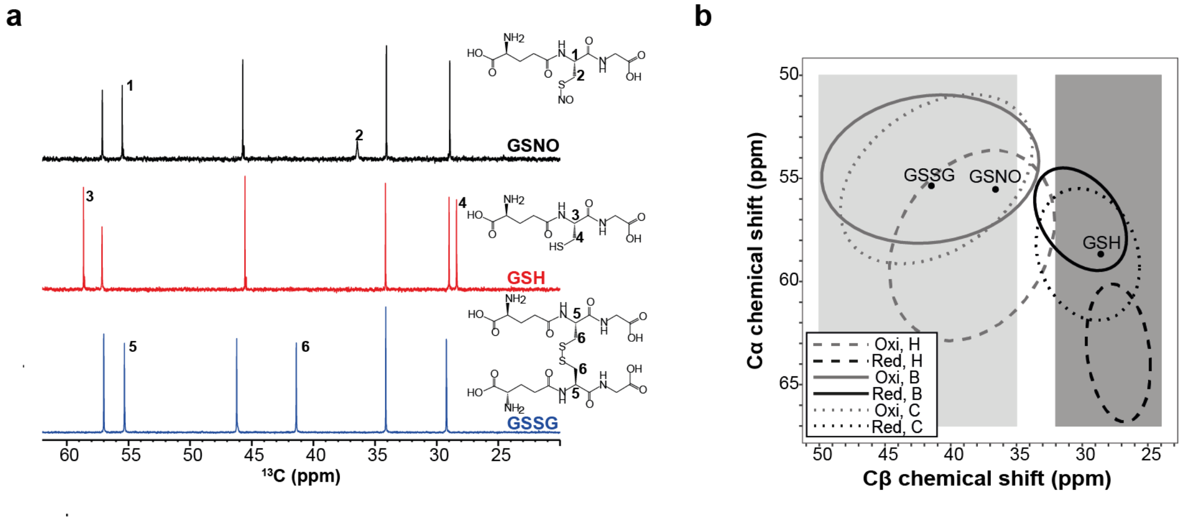

Supplementary Figure 11: Chemical shifts of cysteine modifications. a) 1D ^{13}C NMR spectrum of S-nitroso glutathione (black), glutathione (GSH) (red) and glutathione disulfide (GSSG) (blue). C_α and C_β chemical of cysteine in odd numbers and even numbers, respectively. (**1**=55.489 ppm, **2**= 36.512 ppm; **3**=58.618 ppm, **4**=28.492 ppm; **5**=55.323 ppm, **6**=41.390 ppm). **b**) C_α and C_β chemical shifts from GSSG, GSNO and GSH compared to values derived from a chemical shift database[24]. Ellipses show 90% of the corresponding chemical shifts. Secondary structures: H = helix, B = β-strand and C=coil. The cross-peak region for oxidized cysteine residues is highlighted in light grey and for reduced cysteine in dark grey.

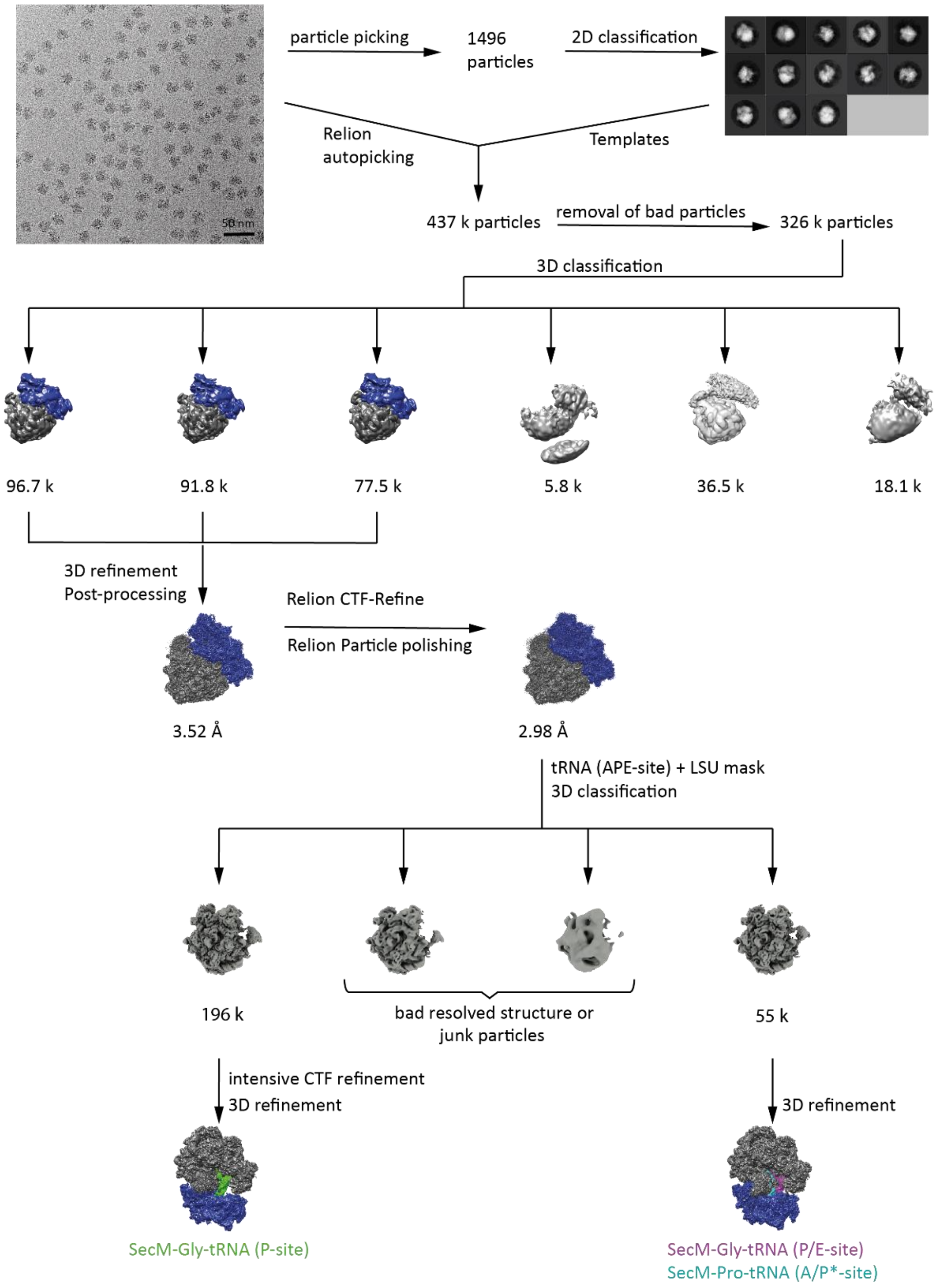

Supplementary Figure 12: Overview of the processing-pipeline for cryo-EM structure of U32SecM. Arrows indicate the direction of processing. The micrograph shows the expected size of the ribosome particles under cryo conditions. 1496 particles were picked manually and were further used to generate 2D classes as template for the auto-picking in Relion. 437 k particles were picked and after several rounds of 2D classification, 326 k good particles remained. After 3D classification three classes with both 50S subunit (grey) and 30S subunit (blue) were used for 3D

refinement and post-processing. The obtained 3.52 Å structure was CTF-refined and polished to a 2.98 Å structure using Relion. All tRNA sites and the LSU were used as a mask for masked 3D classification. Four distinct classes were obtained, with one class showing clear P-site tRNA, one class showing P/E-site and two classes with low resolved structure or junk. The class with tRNA density in the P-site was intensively CTF refined and both classes with clear tRNA density were 3D refined and post-processed using Relion. The final resolution was 2.58 Å for the ribosome with P-site tRNA (green) and 3.19 Å for the ribosome with P/E-site tRNA (pink) and A/P*-site tRNA (cyan).

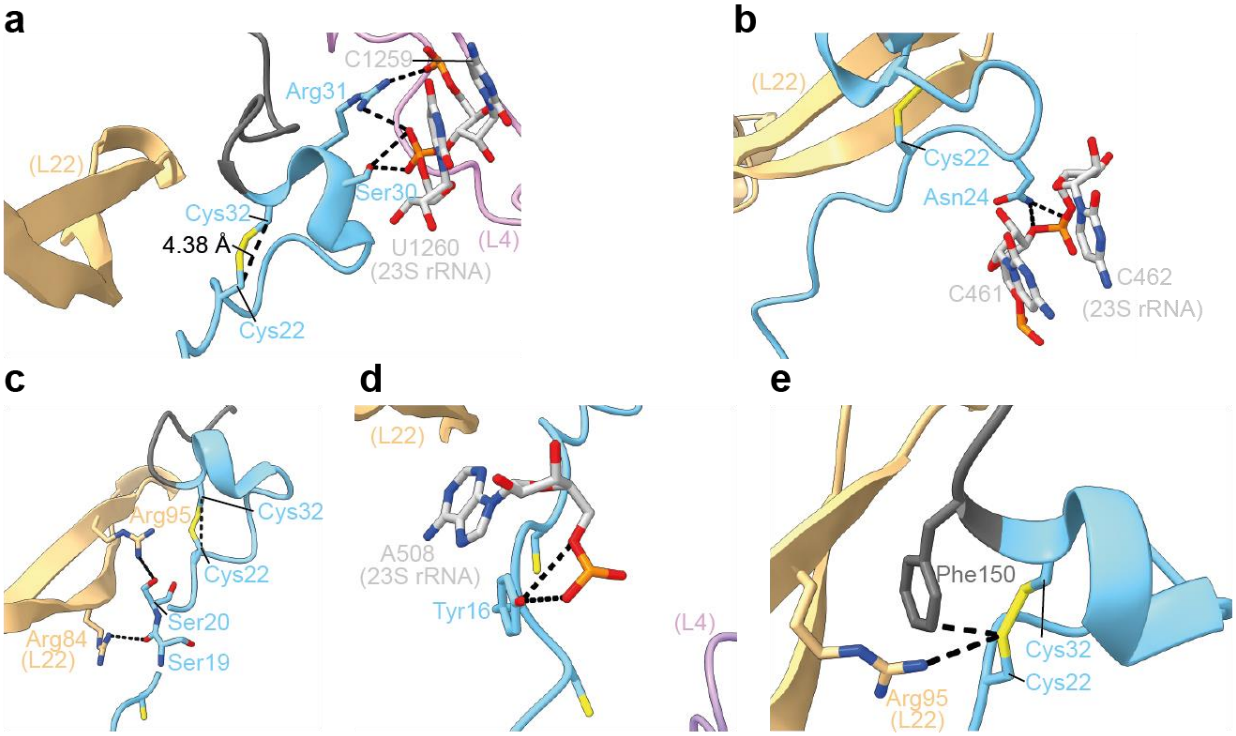

Supplementary Figure 13: **Interactions between ribosomal exit tunnel and U32SecM NC. a)** Interaction of GBC residues Arg31(light blue) with C1259 and U1260 of 23S rRNA and Ser30 with U1260. **b)** Hydrogen bonding between Asn24 side chain of GBC with phosphate backbone of C461 and C462 (light grey). **c)** Interaction between Ser20 and Ser19 of GBC with Arg95 and Arg84 of L22, respectively. **d)** Hydrogen bonding between Tyr16 of GBC and the phosphate backbone of A508 of the 23S rRNA. **e)** Close proximity of Cys22 to Phe150 of SecM and Arg95 of L22. L4 in light pink, L22 in light orange, L23 in light purple, 23S rRNA in light grey, SecM in dark grey and

GBC in light blue. Nucleotides and amino acids are shown in one letter and three letter code, respectively.

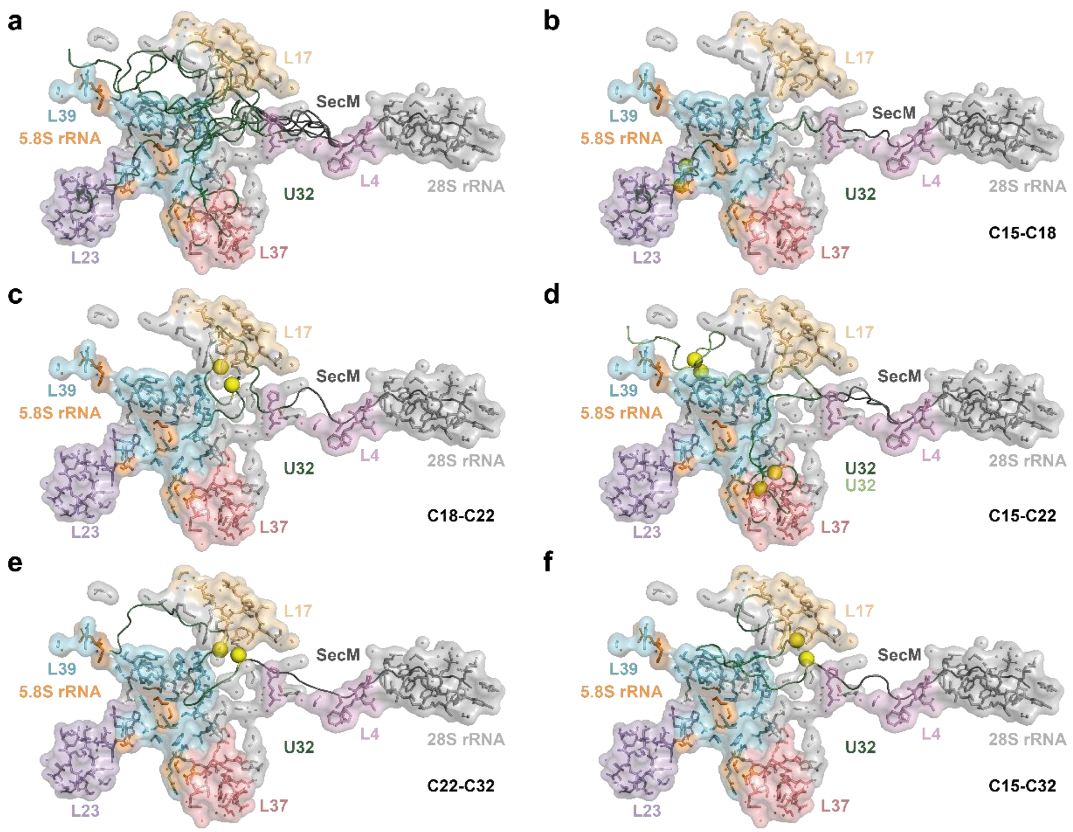

Supplementary Figure 14: Simulation of U32SecM within the ribosomal exit tunnel of *H. sapiens* using flexible-meccano. a) Ensemble of structures within the tunnel. U32SecM chains with Cys15-Cy18 **(b)**, Cys18-Cys22 **(c)**, Cys15-Cys22 **(d),** Cys22-Cys32 **(e)** and Cys15-Cys32 **(f)** in close proximity for possible disulfide bridge. Cysteine residues are highlighted with a yellow ball. SecM in dark grey, U32 in green and lightgreen. Ribosomal proteins L4 (light pink), L17 (light orange) and L23 (light violet), L37 (salmon), L39 (cyan), 5.8S rRNA (orange) and 28S rRNA (light grey) shape the ribosomal tunnel. The ribosomal protein L17 of *H. sapiens* is the structural

equivalent to L22 in *E. coli* ribosomes. The simulation included residues from Asp8 in GBC to Trp155 in SecM. The tunnel dimensions of PDB 4UG0 were used.
