# Supplementary material for: Cysteine oxidation and disulfide formation in the ribosomal exit tunnel
Source: Nat Commun. 2020 Nov 4;11:5569. doi: 10.1038/s41467-020-19372-x (PMC7642426; doi:10.1038/s41467-020-19372-x)
Supplement: Supplementary file 2 — Reporting Summary [file 41467_2020_19372_MOESM2_ESM.pdf]

nature research

Corresponding author(s): Harald Schwalbe

Last updated by author(s): Sep 21, 2020

Reporting Summary

Nature Research wishes to improve the reproducibility of the work that we publish. This form provides structure for consistency and transparency in reporting. For further information on Nature Research policies, see our Editorial Policies and the Editorial Policy Checklist.

Statistics

For all statistical analyses, confirm that the following items are present in the figure legend, table legend, main text, or Methods section.

n/a	Confirmed	
☐	☒	The exact sample size (*n*) for each experimental group/condition, given as a discrete number and unit of measurement
☐	☒	A statement on whether measurements were taken from distinct samples or whether the same sample was measured repeatedly
☒	☐	The statistical test(s) used AND whether they are one- or two-sided<br>*Only common tests should be described solely by name; describe more complex techniques in the Methods section.*
☒	☐	A description of all covariates tested
☒	☐	A description of any assumptions or corrections, such as tests of normality and adjustment for multiple comparisons
☐	☒	A full description of the statistical parameters including central tendency (e.g. means) or other basic estimates (e.g. regression coefficient) AND variation (e.g. standard deviation) or associated estimates of uncertainty (e.g. confidence intervals)
☒	☐	For null hypothesis testing, the test statistic (e.g. F, t, r) with confidence intervals, effect sizes, degrees of freedom and P value noted<br>*Give P values as exact values whenever suitable.*
☒	☐	For Bayesian analysis, information on the choice of priors and Markov chain Monte Carlo settings
☒	☐	For hierarchical and complex designs, identification of the appropriate level for tests and full reporting of outcomes
☒	☐	Estimates of effect sizes (e.g. Cohen's d, Pearson's r), indicating how they were calculated

Our web collection on statistics for biologists contains articles on many of the points above.

Software and code

Policy information about availability of computer code

Data collection	Topspin, SerialEM
Data analysis	R-4.0.2, Topsin3.5-4.08, Cara, Sparky, COOT, PHENIX, USCF Chimera, USCF ChimeraX, Pymol, Relion, MotionCor2, CTFFIND4.1, MetaMorpheus, Flexible-meccano

For manuscripts utilizing custom algorithms or software that are central to the research but not yet described in published literature, software must be made available to editors and reviewers. We strongly encourage code deposition in a community repository (e.g. GitHub). See the Nature Research guidelines for submitting code & software for further information.

Data

Policy information about availability of data

All manuscripts must include a data availability statement. This statement should provide the following information, where applicable:

- Accession codes, unique identifiers, or web links for publicly available datasets
- A list of figures that have associated raw data
- A description of any restrictions on data availability

PDB 3JBV and 3JBU (Electron Microscopy Data Bank under accession code of 6483 and 6486) were used for 3D classification and model building. Data that support the finidings of this study have been deposited in the Protein Data Bank (PDB)with the accession codes 6QDW and 6YSE and in the Electron Microscopy Data Bank (EMDB) under the accession codes 4531 and 10891.
The data set from Wang, C.-C., Lai, W.-C. & Chuang, W.-J. Predicting the redox state and secondary structure of cysteine residues in proteins using multi-dimensional classification of NMR chemical shifts. J. Biomol. NMR 66, 55–68 (2016) (DOI: 10.1007/s10858-016-0057-6) from the Biological Magnetic Resonance Data Bank (BMRB) was used to draw the ellipsis in the Cα-Cβ plots. Each ellipse contains 90% of the corresponding secondary structure.

April 2020

The following figures contain raw data.
- Figure 1, 2, Supplementary Figure 2, 3, 4, 7, 8, 9
Source data are provided with this paper.

Field-specific reporting

Please select the one below that is the best fit for your research. If you are not sure, read the appropriate sections before making your selection.

☒ Life sciences ☐ Behavioural & social sciences ☐ Ecological, evolutionary & environmental sciences

For a reference copy of the document with all sections, see nature.com/documents/nr-reporting-summary-flat.pdf

Life sciences study design

All studies must disclose on these points even when the disclosure is negative.

Sample size	One protein sample (2-3.5 nmol) from one purification procedure was measured by solid-state NMR, except for U32SecM as listed in the manuscript (2 samples). For U32SecM C18 and U43SecM unlabeled and 13C, 15N labeled cysteine samples were measured by LC-MS/MS. All other samples measured using LC-MS/MS were 13C, 15N cysteine labeled.<br>The Relative Integration Ratio was determined using four slightly different integration areas as listed in the manuscript.
Data exclusions	No data was excluded
Replication	Except for U32SecM which was prepared with a higher oxidation ratio as well, one protein sample per constructs was prepared.The U32SecM C18 and U43SecM constructs were further prepared in unlabeled medium for LC-MS/MS experiments. Protein expression and purification is reproducible. The oxidation level of the individiual samples were measured once (except U32SecM) due to the long sample preparation and measurement time. Oxidation was recorded in all samples, 13C, 15N cysteine labeled and unlabeled.
Randomization	Not relevant for this work as no clinical study was executed.
Blinding	Not relevant for this work as no clinical study was executed.

Reporting for specific materials, systems and methods

We require information from authors about some types of materials, experimental systems and methods used in many studies. Here, indicate whether each material, system or method listed is relevant to your study. If you are not sure if a list item applies to your research, read the appropriate section before selecting a response.

Materials & experimental systems

n/a	Involved in the study	
☐	☒	Antibodies
☒	☐	Eukaryotic cell lines
☒	☐	Palaeontology and archaeology
☒	☐	Animals and other organisms
☒	☐	Human research participants
☒	☐	Clinical data
☒	☐	Dual use research of concern

Methods

n/a	Involved in the study	
☒	☐	ChIP-seq
☒	☐	Flow cytometry
☒	☐	MRI-based neuroimaging

Antibodies

Antibodies used	Nonoclonal Anti-polyhistidine antibody produced in mouse. Merck (Darmstadt, Germany) H1029.<br>https://www.sigmaaldrich.com/content/dam/sigma-aldrich/docs/Sigma/Datasheet/2/h1029dat.pdf
Validation	https://www.sigmaaldrich.com/sapfs/PROD/sap/certificate_pdfs/COFA/Q14/H1029-100UL0000093768.pdf<br>Antibody was tested on uninduced cell lysate and flow-thorugh ribosomes as negative control and on different proteins containing a 6x or 10x His-tag as positive control.
